# Evidence of peripheral olfactory impairment in the domestic silkworms: insight from the comparative transcriptome and population genetics

**DOI:** 10.1186/s12864-018-5172-1

**Published:** 2018-11-01

**Authors:** Chuan-Zhen Qiu, Qiu-Zhong Zhou, Ting-Ting Liu, Shou-Min Fang, Ya-Wang Wang, Xin Fang, Chun-Li Huang, Quan-You Yu, Chun-Hong Chen, Ze Zhang

**Affiliations:** 1 School of Life Sciences, Chongqing University, Chongqing, 401331 China; 2 College of Life Science, China West Normal University, Nanchang, 637002 China; 3 Agriculture and Food, CSIRO, Acton, 2601 Australia

**Keywords:** Antennae, Transcriptome, Olfactory adaptation, Functional constraint, Silkworm

## Abstract

**Background:**

The insect olfactory system is a highly specific and sensitive chemical detector, which plays important roles in feeding, mating and finding an appropriate oviposition site. The ecological niche of *Bombyx mori* has changed greatly since domestication from *B. mandarina*, and its olfactory response to environmental odorants clearly decreased. However, the mechanisms that result in the olfactory impairment are largely unknown.

**Results:**

The antennal transcriptomes were compared between the domestic and wild silkworms. Comparison of the same sex between the domestic and wild silkworms revealed 1410 and 1173 differentially expressed genes (DEGs) in males and females, respectively. To understand the olfactory impairment, we mainly focused on the olfactory-related genes. In total, 30 olfactory genes and 19 odorant-degrading enzymes (ODEs) showed differential expression in the two comparisons, in which 19 and 14 were down-regulated in the domestic silkworm, respectively. Based on population genomic data, the down-regulated odorant receptors (ORs) showed a higher ratio of unique non-synonymous polymorphisms to synonymous polymorphisms (*N/S* ratio) in the domestic populations than that in the wild silkworms. Furthermore, one deleterious mutation was found in OR30 of the domestic population, which was located in transmembrane helix 6 (TM6).

**Conclusions:**

Our results suggested that down-regulation of the olfactory-related genes and relaxed selection might be the major reasons for olfactory impairment of the domestic silkworm reared completely indoor environment. Reversely, wild silkworm may increase expression and remove deleterious polymorphisms of olfactory-related genes to retain sensitive olfaction.

**Electronic supplementary material:**

The online version of this article (10.1186/s12864-018-5172-1) contains supplementary material, which is available to authorized users.

## Background

The olfactory system is mainly responsible for the sense of smell. Insects rely on a wide range of olfactory senses to locate mates, find an appropriate oviposition site as well as to avoid predators and other dangers. Insect antenna is served as an important periphery olfactory system, which contained several sensillum types, such as sensilla trichodea (medium-length and long), sensilla basiconica and sensilla coeloconica [1]. It plays an improtant role in reception and processing of semiochemicals, and rapid inactivation of the odorants once they have conveyed information [2].

Odor perception is a selective and sensitive process, which is heavily dependent on the various receptors expressed on olfactory sensory neurons (OSNs) in antennae [2, 3]. Olfactory receptors include odorant receptors (ORs), ionotropic receptors (IRs), and sensory neuron membrane proteins (SNMPs). In insects, odor molecules are first captured and transported to the receptors by water-soluble extracellular proteins that are located in the fluid surrounding the sensory dendrite of antennal sensilla, including odorant-binding proteins (OBPs) and chemosensory proteins (CSPs) [4]. Once the message is conveyed, the chemical signal would be rapidly inactivated to prevent the accumulation of residual stimulant [2]. Those antenna-biased odorant-degrading enzymes (ODEs) play important roles in inactivating sex pheromones and plant volatile elicitors [5, 6]. Thus, olfaction is orchestrated by a series of olfactory proteins and odorant-degrading enzymes.

*Bombyx mori* is one of the model organisms for the study of insect olfaction, especially in pheromone perception [7, 8] and functional diversities of odorant receptors [9, 10]. About 5000 years ago, *B. mori* was domesticated from the wild silkworm *B. mandarina* [11]. It was found that the domestic silkworm’s response to environmental odorants was clearly decreased when compared with the wild silkmoth [1]. Previous studies suggested that high olfactory sensitivity has metabolic costs and would be a waste of energy if the organism’s physiology were not ready for the final behavioral output [12]. In the wild silkmoth, maintaining olfactory sensitivity should be an important evolutionary strategy for survival and reproduction. The comparison between *B. mori* and its wild counterpart might be interesting for studying environment-dependent olfactory adaptation.

To cope with the variable conditions, insect OSNs can adapt to their environment through olfactory plasticity [12]. Some studies have demonstrated that reduced expression of olfactory genes can lower the sensitivity of a sensory neuron [13–15]. In *Culex quinquefasciatus*, silencing the *CquiOBP1* gene showed significantly lower electrophysiological responses to known mosquito oviposition pheromones than the antennae of water-injected, control mosquitoes [14]. In this regard, expression plasticity of olfactory genes might be an important reason for the variation of olfaction phenotype. In addition, genetic variation of olfactory genes is another mechanism for altering the sensitivity of OSNs. In *Drosophila melanogaster*, single-nucleotide polymorphisms (SNPs) in OBP genes contribute to individual variation in chemosensory behavior [16, 17]. Furthermore, olfactory genes may have undergone different selective pressures in domestic and wild populations [18]. To retain sensitive olfaction, evolutionary processes observed in the wolf have had the effect of removing deleterious polymorphisms and accumulating tolerant polymorphisms in olfactory genes, when compared with dogs [18].

In this study, we analyzed the antennal transcriptomes of the domestic and wild silkmoths, and mainly focused on gene families that have been implicated in olfaction. Furthermore, the genomes of eight domestic silkworms and seven wild silkworm samples have been resequenced in our lab. These genomic data enable us to detect evolutionary rates and deleterious mutations of differentially expressed chemosensory genes. The objective of this study was to detect expression difference of olfactory-related genes in the antennae of the domestic and wild silkworms and to deepen understanding of how the domestic silkworms to impair olfactory sensitivity. Comparison of the indoor species and its corresponding wild species would help us understand the potential mechanisms of olfactory adaptation in wild condition.

## Materials and methods

### Silkworm collection and sample preparation

The domestic silkworm strain *Dazao* was reared on fresh mulberry leaves at 25 ± 1 °C and 75% ± 3% relative humidity (14 h lights : 10 h dark) in an indoor chamber. The wild silkworms were collected from Chongqing City, China. The larvae of the wild silkworms were reared on mulberry trees in open-air field chambers. Two days after pupation, the pupae of the domestic and wild silkworms were transferred to a same indoor chamber until dissection of adult antennae. The intact antennae of 30 single-sex individuals were dissected from virgin moths at 24 h after adult eclosion and used for one sample. Two replicates were taken for the antennae of female (W_F) and male (W_M) of the wild silkworm, female (D_F) and male (D_M) of the domestic silkworm, respectively. All the samples were preserved in RNAlater (Ambion, Austin, USA) and stored at − 80 °C for RNA isolation.

### RNA sequencing and assembly

RNA isolation, library preparation, and sequencing. Total RNA of the antennae was extracted with Trizol reagent (Invitrogen, Burlington, ON, Canada) according to the manufacturer’s instructions. RNA purity was checked with the NanoPhotometer spectrophotometer (IMPLEN, Westlake Village, CA, USA). The quality of the RNA samples was checked using an Agilent Bioanalyzer 2100 (Agilent Technologies, Santa Clara, CA, USA). Illumina mRNA sequencing libraries were run for paired-end reads sequencing on an Illumina HiSeq™ Genome Analyzer platform (Novogene, Beijing, China).

Quality control and assembly. The raw reads were filtered by removing adaptor sequences and low-quality sequences containing > 10% poly-N or > 50% of bases whose Phred quality scores ≤5 with NGS QC Toolkit v2.3.3 [19]. The reference genome of *B. mandarnia* is unavailable. The reads of the whole genome and transcriptome sequencing were often mapped to the domestic silkworm reference genome [20, 21]. The index of silkworm reference genome (http://silkworm.genomics.org.cn/) was built using Bowtie version 2.2.8 [22]. The clean reads of *B. mori* and *B. mandarnia* were aligned to the reference genome using TopHat version 2.1.1 [23]. The uniquely mapped reads were retained and used for further analysis. Finally, transcripts were assembled by Cufflinks version 2.2.1 [24].

### Identification of differentially expressed genes (DEGs)

Gene expression levels were estimated using FPKM (fragments per kilobase of transcript per million mapped reads) values with Cufflinks [24]. HTSeq v0.5.4 [25] was used to count the number of reads mapped to each gene. Identification of DEGs was performed using the DESeq package [26]. The *P*-value adjusted by Benjamini-Hochberg method of 0.05 and fold-change of 3 was set as the threshold values for significant differential expression.

### GO annotation and KEGG enrichment analyses of DEGs

To predict the function, all the unigenes were used to BLASTX search against the nr (non-redundant) protein database in NCBI with an *E*-value <1e-5. The BLASTX results were converted into functional annotations by gene ontology (GO) terms using Blast2GO software [27]. The statistical significance of the functional GO enrichment was evaluated using a false discovery rate (FDR < 0.05). Pathways from Kyoto Encyclopedia of Genes and Genomes (KEGG) were assigned using the online KEGG Automatic Annotation Server (KAAS).

### Identification of novel olfactory-related genes

All the antenna-expressed genes (FPKM ≥1 in at least one of the four antennal samples) were translated using TransDecoder (http://transdecoder.github.io) assisted by Pfam domain information. Hidden Markov Model (HMM) files were downloaded from Pfam database (http://pfam.xfam.org/), including iGluR ligand-gated ion channel (PF00060), odorant receptor (PF02949/PF13853). HMM files were used to screen the translated proteins of the antenna-expressed genes using HMMER 3.0 (*E*-value <1e-5). Using known olfactory-related protein sequences as queries, BLASTP was also used to search against the predicted protein database of the antenna-expressed genes (*E*-value <1e-5). All the significant hits were subsequently checked by BLASTP against nr database in NCBI.

The putative amino acid sequences of ORs and IRs were aligned using MUSCLE [28]. Positions that had a high percentage of gaps (> 70%) were trimmed. The VT + G + F was selected as the most suitable model of evolution by ProtTest 3.2 [29] based on the Akaike information criterion (AIC). Maximum-likelihood (ML) trees were reconstructed using RAxML version 8.2.12 [30] with “PROTGAMMAVTF” implementation, four discrete rate categories, and 100 bootstrap replicates. The neighbor-joining (NJ) and maximum parsimony (MP) trees were reconstructed by MEGA X [31]. NJ trees were reconstructed with 500 bootstrap replicates, Jones–Taylor–Thornton (JTT) model, and a gamma distribution (shape parameter = 2.21), which were identified as relatively good models by ProtTest. MP trees were carried out with default settings, namely: the subtree pruning and regrafting (SPR) algorithm, random addition of sequences with ten replicates, and bootstrap test with 500 replicates. Based on the phylogenetic trees, novel IR genes were named according to a unified nomenclature system and Croset’s method [32].

### Population genetics and molecular evolution of the olfactory genes

In this study, eight domestic silkworm individuals and seven wild silkworms were used for whole-genome sequencing with Illumina’s HiSeq 4000 system. The domestic samples were from eight silkworm strains, *7532*, *S03*, *S02*, *Xianghui*, *HB05*, *Yanjinhuang*, *Xiaoshiwan*, and *Jianpuzhai*. One individual per geographical location in China was captured, including Beibei District in Chongqing City, Hongya County in Sichuan Province, Anyue County in Sichuan Province, Nanchong City in Sichuan Province, Ziyang City in Sichuan Province, Wuhan City in Hubei Province, and Suzhou City in Jiangsu Province. The clean reads of each sample were mapped to the silkworm reference genome using BWA (https://sourceforge.net/projects/bio-bwa/). Picard (http://broadinstitute.github.io/picard/) was used for sorting the BAM file by reading position and removing the highly repetitive reads. The single nucleotide polymorphism (SNP) and INDEL in each sample were identified by GATK v2.7 [33], Samtools [34] and FreeBayes [35]. The overlapped SNPs and INDELs detected by the three tools were used for further analysis. The consensus sequences of the candidate genes were extracted in the genome sequence of each sample, including the coding sequences (CDS) and gene sequences comprised of CDS, introns, and 2-Kb 3′ and 5′ flanking regions.

In the domestic and wild populations, genetic diversities (π) of the differentially expressed olfactory genes were estimated by DnaSP 5.1 [36]. Tajima’s D, Fu, and Li’s D*, and Fu and Li’s F* tests were used to detect whether the genes were evolving neutrally using DnaSP. The ratios of non-synonymous (*d*_*N*_) and synonymous (*d*_*S*_) substitution rates were calculated by the YN00 program implemented in the PAML 4.5 package [37]. In addition, we counted the numbers of non-synonymous (N) and synonymous (S) SNPs unique to the domestic and wild populations, respectively. And then, the population-unique *N*/*S* ratios were calculated for the differentially expressed olfactory genes.

### Quantitative real-time PCR (qPCR)

The qPCR validation experiment was performed, which the method was introduced in our previous study [38]. The primers and annealing temperature of each gene were listed in Additional file 1: Table S1. Gene expression levels were normalized against the corresponding ribosomal protein L3 (*RpL3*) expression levels. The relative expression level of each gene was calculated by the relative quantification (*R* = 2^-ΔΔCt^) method [39].

## Results

### Overview of the antennal transcriptomes in the silkworms

To explore the mechanisms of olfactory adaptation, we collected the intact antennae from the domestic silkworm strain *Dazao* and wild silkworm. The RNA of each sample from 30 individuals was sequenced using an Illumina Genome Analyzer (II). After filtering low-quality reads and trimming adapters (Additional file 2: Table S2), the clean reads were mapped to the *B. mori* reference genome (Additional file 3: Table S3). All of the mapped reads were merged and assembled using Cufflinks [24]. Totally, 22,767 unigenes were assembled from the antennal transcriptomes of the domestic and wild silkworms (Additional file 4: Table S4). To discard transcript models that had no read coverage or low coverage (FPKM < 1) in all samples, transcripts with FPKM ≥1 in at least one sample were considered for expression. The number of genes with FPKM ≥1 varied from 13,080 to 14,022 among the four samples (Additional file 5: Table S5). KEGG assignments were used to classify the functions of the antenna-expressed genes. Relatively, signal transduction has a higher gene number (*n* = 550) than the other pathways (Additional file 6: Figure S1). In addition, we found some pathways related to olfactory and detoxification functions, such as the sensory system (*n* = 56), xenobiotics biodegradation and metabolism (74), and environmental adaptation (*n* = 61). In the antennae, we found that 61–74 genes were highly expressed (FPKM > 1000), in which 46 genes were overlapped among the four samples (Additional file 5: Table S5). In addition, six genes (*BmGOBP1*, *BmGOBP2*, *BmOBP27*, *BmPBP1*, *BmOBP20*, and *BmCSP1*) were extremely highly expressed (FPKM > 10,000) in all the four samples (Additional file 4: Table S4). These results were in accord with the olfactory function of insect antennae.

### Identification and expression of olfactory-related genes in the antennal transcriptomes

**Fig. 1:**
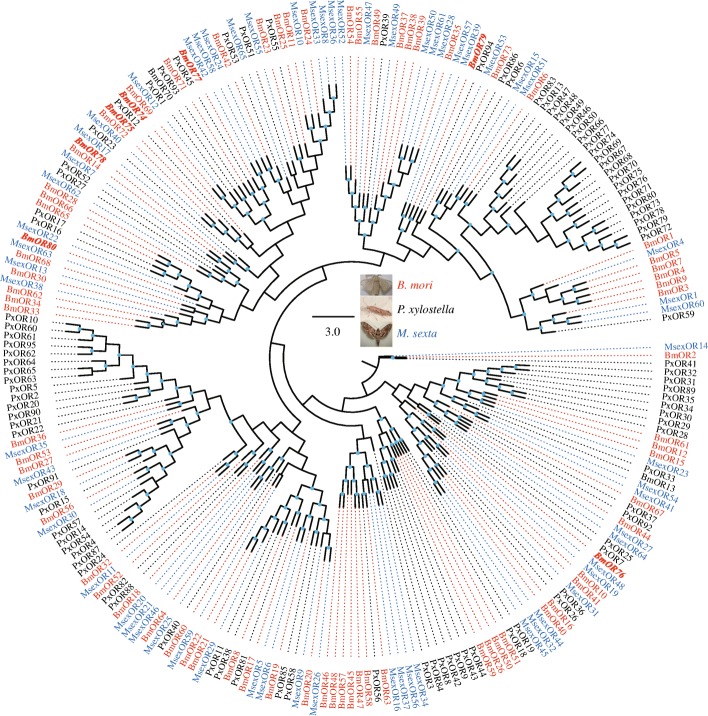
Maximum-likelihood phylogenetic tree of ORs in *B. mori*, *Plutella xylostella*, and *Manduca sexta*. Bootstrap values > 50% have doted on the nodes. The novel ORs of *B. mori* (BmORs) are in italic and bold. The other OR sequences of *B. mori*, *P. xylostella* (PxORs) and *M. sexta* (MsORs) were from the previous studies [59, 60]

**Fig. 2:**
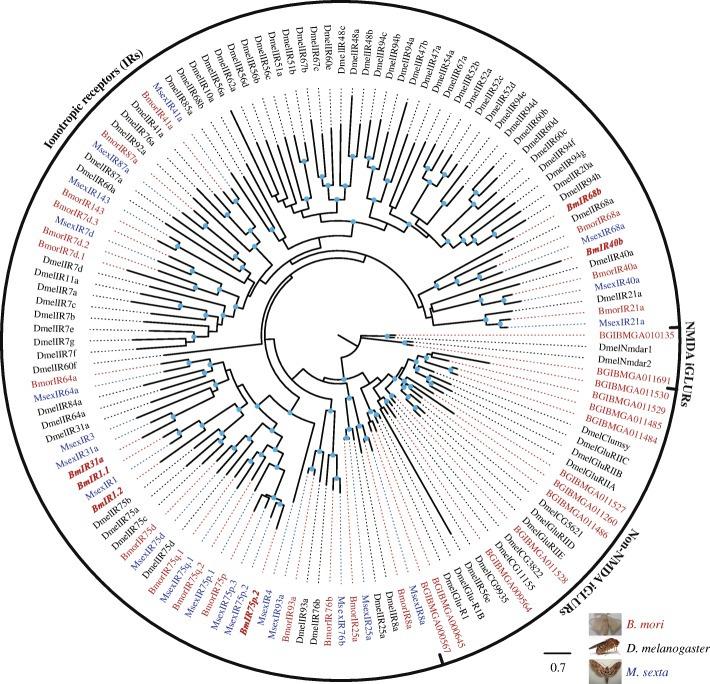
ML tree of IRs in *B. mori*, *D. melanogaster*, and *M. sexta*. Bootstrap values > 50% have doted on the nodes. The novel BmIRs were in italic and bold. The known IRs and iGLURs of *B. mori*, *M. sexta*, *D. melanogaster* were obtained from previous studies [59]

In the silkworm, olfactory-related gene families have been widely annotated in the whole genome. Using the known gene sequences, BLAST and HMMER searches were used to identify novel olfactory-related genes in the assembled unigenes of the antennal transcriptomes (Table 1). Seven novel OR genes were characterized and assigned new names numbering from 74 upwards to 80 (Fig. 1, Additional file 7: Table S6). Ionotropic receptor (IR) is a new family of olfactory receptors and a variant subfamily of iGluRs [40]. In the previous study, 18 IR members were identified in the silkworm genome [32]. In this study, six novel ionotropic receptors were characterized (Fig. 2, Additional file 7: Table S6). In insects, some important gene families related to odorant degrading function were revealed, such as carboxylesterases (COEs), cytochrome P450 monooxygenases (P450s), glutathione S-transferases (GSTs), aldehyde oxidases (AOXs), and UDP-glucuronosyltransferases (UGTs) [41]. These important families were detected in this study, and only few novel genes were identified for COEs (1) and P450s (1) (Table 1).

**Table 1:** Olfactory-related gene families and the number of genes expressed in the antennae

Olfactory-related genes	Genome annotation	Novel gene	D_M	D_F	W_M	W_F
Olfactory genes						
Odorant binding proteins (OBPs)	44	2	30	30	30	32
Odorant receptors (ORs)	71	7	42	44	51	51
Sensory neuron membrane proteins (SNMPs)	2	0	2	2	2	2
Ionotropic receptors (IRs)	18	6	12	14	15	16
Chemosensory proteins (CSPs)	22	0	20	18	19	18
Potential ODEs						
Glutathione S-transferases (GSTs)	23	0	18	19	20	20
UDP-glycosyltransferases (UGTs)	45	0	18	19	21	21
Carboxylesterases (COEs)	76	1	34	38	36	36
Cytochrome P450 (P450s)	84	1	29	31	38	39
Aldehyde oxidases (AOXs)	6	0	3	3	3	4

Genes with FPKM ≥1 in at least one of the four antennal samples were considered to be expressed. The genome-annotated genes were retrieved from the previous studies, including OBPs [58], ORs [59, 60], SNMPs [42], IRs [59], CSPs [61], GSTs [62], UGTs [63], COEs [64], P450s [65], and AOXs [66]. Novel genes were identified in transcriptome assembly, and its corresponding sequences were included in Additional file 7: Table S6

Our results indicated that more than half of the whole-genome OR, IR, and OBP genes were expressed (FPKM ≥1) in the antennae of the domestic and wild silkworms (Table 1). Two sensory neuron membrane protein (SNMP) genes were identified in the domestic silkworm genome [42], and both of them were expressed in the adult antennae (Table 1). In addition, almost all of the CSPs showed expression signals in the antennae. For the five potential ODE families, nearly three-quarters of the GSTs, almost half of the UGTs, COEs, and P450s and 4 out of 6 AOXs were shown to be expressed. Within the ten olfactory-related families of interest here, more than 60% of the antennal-expressed OBPs, CSPs and AOXs showed high expressions (FPKM > 100) (Additional file 4: Table S4). In contrast, about 60% of the antenna-expressed IRs, ORs, and COEs and about 40% of P450 and UGTs genes presented low expressions (FPKM < 10) in all the four samples.

### Identification of DEGs and primary candidates related to olfactory impairment

**Fig. 3:**
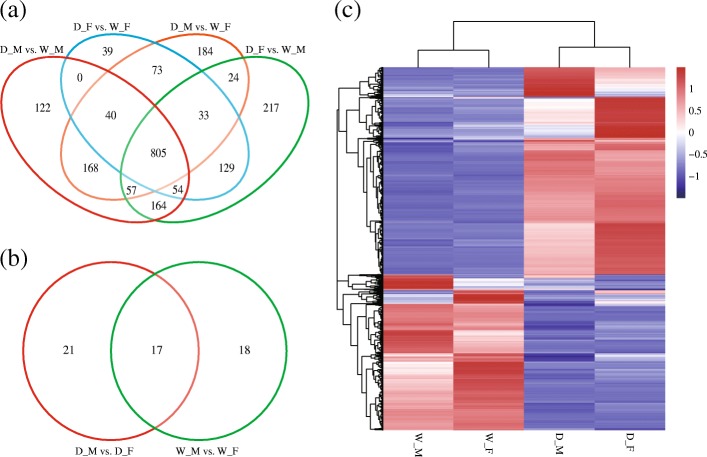
Hierarchical clustering and Venn diagram of the differentially expressed genes in the antennal transcriptome of the domestic and wild silkworms. **a** Venn diagram showing the number of the DEGs between the domestic and wild silkworms. **b** Venn diagram of the DEGs in D_M vs. D_F and in W_M vs. W_F. **c** Hierarchical clustering of all the differentially expressed genes. The mean values of FPKM from two biological replicates were used for hierarchical clustering using hcluster algorithm (http://www.omicshare.com/tools). The blue bands indicate low expressions; the red bands indicate higher expressions

**Fig. 4:**
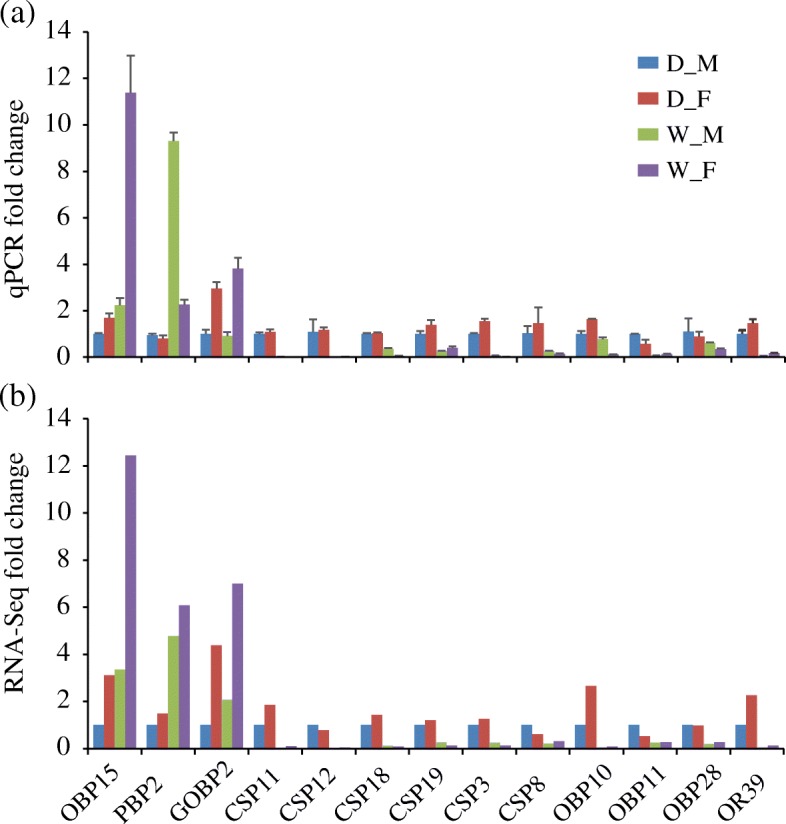
Validation of the DEGs by qPCR method. **a** Quantitative real-time PCR (qPCR) results. The relative expression of candidate genes was normalized against *RpL3*. The fold-change of each gene was calculated by dividing the relative expression level in D_M. SEM (the standard error of the mean) error bars are showed. **b** RNA sequencing results. The Y-axis indicates the fold change of FPKM compared with the corresponding FPKM values in D_M

For a more global view, the differentially expressed genes of all comparisons were identified by DESeq [26]. From the total of 22,767 unigenes, we identified 2197 DEGs among the four samples (Additional file 8: Table S7). The numbers of DEGs among the six comparisons are shown in Fig. 3a and b. Based on FPKM values, hierarchical clustering of all the DEGs was conducted, which indicated that more genes were up-regulated in the domestic silkworm (Fig. 3c). To validate the RNA-seq data, qPCR was performed for 13 DEGs related to olfaction. The results of qPCR and Illumina sequencing data were consistent with each other (Fig. 4). In order to understand the functions of the DEGs, GO enrichment analysis was performed in BLAST2GO [27]. We found that some GO terms were related to olfaction (Additional file 9: Figure S2; Additional file 10: Table S8), such as sensory perception (GO:0007600; *n* = 49) and odorant binding (GO:0005549; *n* = 58).

**Fig. 5:**
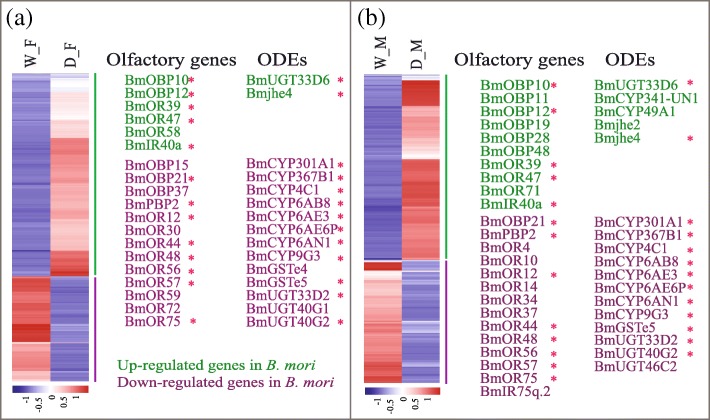
Olfactory-related genes showed differential expressions in the same sex between the domestic and wild silkworms. **a** Hierarchical clustering of the DEGs and differentially expressed olfactory and putative ODE genes in females. **b** Hierarchical clustering of the DEGs and differentially expressed olfactory-related genes in males. The shared genes between the two comparisons were added stars (*) at the end of gene names

To understand the olfactory impairment, comparison of the same sex between the domestic and wild silkworms may be more meaningful. Totally, 1410 and 1173 DEGs were identified in D_M vs. W_M and D_F vs. W_F, respectively (Additional file 8: Table S7). GO enrichment analysis suggested that metabolic process and catalytic activity categories represent the highest numbers within the DEGs (Additional file 10: Table S8). Through the functional annotations based on GO and BLAST homology searches, except for the olfactory-related genes, no more interesting gene types were found. Thus, we only focused on the olfactory-related genes in further analysis. Compared with the domestic silkworms, 13 out of 19 olfactory genes and 13 out of 15 putative ODEs were up-regulated in females of the wild silkworm (Fig. 5a), 14 out of 24 olfactory genes and 12 out of 17 ODEs were up-regulated in males (Fig. 5b). In the comparisons of the same sex between the domestic and wild silkworms, overall 30 olfactory genes and 19 ODEs were differentially expressed, in which 19 olfactory genes and 14 ODEs were up-regulated in the wild silkworm. This indicated that the decreased expressions of the olfactory-related genes may lower the sensitivity of OSNs in the domestic silkworms [13, 14].

### Molecular population genetics of the olfactory genes down-regulated in the domestic silkworm

Compared with the wild silkworm, the down-regulated olfactory genes (*n* = 19) might be the primary candidates for impairing olfactory sensitivity to plant volatiles in *B. mori*, in which 18 genes belonged to ORs and OBPs (Fig. 5). To further understand olfactory impairment, molecular evolution of the down-regulated ORs and OBPs was investigated. Based on the resequencing genome data of eight domestic silkworm strains and seven wild silkworm samples in our lab, gene sequences were obtained for four OBPs and 13 ORs except for pseudogene OR48 [9]. For the coding sequences (CDS) and whole gene sequences, almost all the genes in the domestic silkworm strains showed lower nucleotide diversities (π) than the wild populations (Table 2). This is in accord with *B. mori* representing a bottlenecked subpopulation of *B. mandarina*. To identify the type of selection that may have occurred in the different groups (domestic, wild silkworms), Tajima’s *D*, Fu, and Li’s D*, and Fu and Li’s F* tests were performed. In the wild population, negative values for almost all of the genes indicated that there were greater numbers of rare alleles. In the domestic strains, most of the differentially expressed olfactory genes contained positive values from those tests, which may be due to a population bottleneck or balancing selection.

**Table 2:** Genetic diversity and neutrality analysis of the ORs and OBPs genes down-regulated in the domestic silkworms

Gene name	CDS length (nt)	*Bombyx mori*							*Bombyx mandarina*						
		No. of strains	CDS		Gene sequence and 2-Kb FR				No. of samples	CDS		Gene sequence and 2-Kb FR			
			π	*d*_*N*_ */d*_*S*_	π	*D*_*T*_	D*	F*		π	*d*_*N*_ */d*_*S*_	π	*D*_*T*_	D*	F*
*OBP15*	366	8	0.0021	0.1135	0.0136	0.6168	0.3254	0.4399	6	0.0063	0.0759	0.0191	−0.3188	− 0.2501	− 0.2914
*OBP21*	627	8	0.0049	0.1650	0.0078	0.3158	0.3451	0.3778	6	0.0061	0.3491	0.0088	−0.0586	− 0.0808	− 0.0837
*OBP37*	330	8	0.0118	0.0420	0.0010	−0.0959	−0.0332	− 0.0533	6	0.0137	0.0297	0.0310	−0.3393	−0.2830	− 0.3247
*PBP2*	372	8	0.0126	0.1672	0.0195	−0.1289	0.0866	0.0403	7	0.0119	0.0846	0.0304	−0.4138	−0.3598	− 0.4128
*OR4*	1275	8	0.0035	0.0773	0.0090	0.6312	0.5437	0.6303	6	0.01	0.1245	0.0149	−0.4607	−0.3944	−0.4496
*OR10*	1167	8	0.0120	0.1913	0.0193	0.0958	0.2275	0.2197	6	0.017	0.1237	0.0271	−0.3930	−0.3541	−0.3990
*OR12*	1179	8	0.0032	0.0731	0.0032	0.1175	0.3814	0.3566	6	0.0089	0.3970	0.0092	−0.6680	−0.6418	−0.7184
*OR14*	1173	8	0.0069	0.0466	0.0119	0.2493	0.3197	0.3388	6	0.0115	0.0712	0.0195	−0.4780	−0.4595	−0.5101
*OR30*	1173	7	0.0110	0.2339	0.0113	−0.1433	−0.1843	− 0.1952	6	0.0146	0.1741	0.0141	0.0283	0.1030	0.0958
*OR34*	762	8	0.0054	0.1868	0.0119	0.1960	0.1716	0.1980	7	0.0075	0.1440	0.0114	−0.4507	−0.3997	− 0.4559
*OR37*	1137	8	0.0031	0.0449	0.0039	0.6891	0.3130	0.4475	6	0.0085	0.2474	0.0083	−0.6435	−0.5994	−0.6693
*OR44*	972	8	0.0046	0.0825	0.0078	0.3408	0.5745	0.5807	6	0.0051	0.0204	0.0168	−0.4299	−0.3900	−0.4387
*OR56*	1206	8	0.0017	0.0905	0.0038	−0.8942	−1.0676	−1.1474	6	0.0166	0.0587	0.0205	−0.5050	−0.4623	− 0.5191
*OR57*	1143	8	0.0027	0.4729	0.0030	−0.8150	−0.8933	− 0.9773	6	0.0012	0.6844	0.0023	0.0001	0.0851	0.0735
*OR59*	984	6	0.0100	0.4959	0.0182	0.1722	0.0275	0.0687	7	0.0066	0.2476	0.0162	−0.3474	−0.3338	− 0.3738
*OR72*	1014	8	0.0080	0.7472	0.0042	−0.9235	−0.8874	−1.0010	6	0.0195	0.6895	0.0075	0.0445	0.0801	0.0798
*OR75*	1158	8	0.0032	0.3597	0.0100	−0.9937	−0.8275	−0.9684	7	0.0099	0.1459	0.0213	−0.4792	−0.4409	− 0.4989

*CDS* coding regions; gene sequence contained coding regions and introns; *2-Kb FR* 2-Kb 3′ and 5′ flanking regions, *NA* not applicable because of zero synonymous differences; π, the average number of nucleotide differences per site between sequences; *D*_*T*_, Tajima’s *D* value; D*, Fu and Li’s D* value; F*, Fu and Li’s F* value

### Evolutionary rate and intolerant mutations of the olfactory genes

**Fig. 6:**
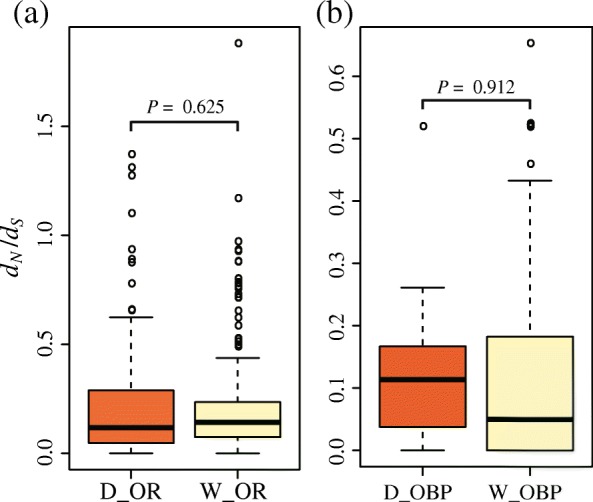
Box plot of the *d*_*N*_/*d*_*S*_ values of the ORs and OBPs down-regulated in the domestic silkworms. **a** The *d*_*N*_/*d*_*S*_ values of the ORs down-regulated in the domestic silkworms. The 13 OR were used for global *d*_*N*_*/d*_*S*_ estimations in PAML (YN00). D_OR: OR genes in the domestic population; W_OR: OR genes in the wild population. **b** The *d*_*N*_/*d*_*S*_ values of the four OBPs down-regulated in the domestic silkworms. D_OBP: OBP genes in the domestic population; W_OR: OBP genes in the wild population

Non-synonymous nucleotide changes do alter protein sequences and could be subject to adaptive selection or relaxation, whereas synonymous changes are more or less neutral. Totally, we identified 463 and 52 synonymous/non-synonymous polymorphism sites for the 13 ORs and 4 OBPs within 15 genome samples, respectively (Table 3). The global *d*_*N*_*/d*_*S*_ ratios were estimated by the YN00 program in the PAML [37]. The results indicated that the *d*_*N*_*/d*_*S*_ ratios of all the 17 olfactory genes were < 1 in the domestic and wild populations (Table 2), suggesting that these genes might have undergone purifying selection. Interestingly, the *d*_*N*_*/d*_*S*_ ratios of ORs and OBPs were different from its corresponding genetic diversities (Table 2), which were comparable in the domestic and wild silkworms (Fig. 6). These results suggested that relaxed purifying selection may be driving the increase of non-synonymous evolutionary rate in the domestic silkworm.

**Table 3:** Numbers of synonymous and non-synonymous nucleotide changes of the ORs and OBPs down-regulated in the domestic silkworms

Polymorphism types	OBPs			ORs		
	N	S	*N/S*	N	S	*N/S*
Polymorphisms across all samples	16	36	0.44	172	291	0.59
Polymorphisms in *B. mori*	9	24	0.38	99	127	0.78
Polymorphisms in *B. mandarina*	13	30	0.43	139	232	0.60
Unique polymorphisms in *B. mori*	3	6	0.50	32	39	0.82
Unique polymorphisms in *B. mandarina*	7	12	0.58	74	162	0.46

*S* synonymous, *N* non-synonymous, *N/S* non-synonymous/synonymous polymorphism ratios; The 4 OBPs and 13 ORs in Table 2 were used for counting the number of synonymous and non-synonymous nucleotide changes

**Fig. 7:**
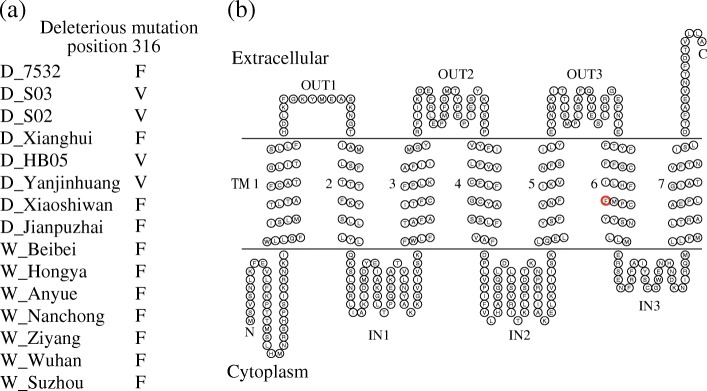
The deleterious amino acid substitution of OR30 in the domestic silkworms. **a** Population-unique deleterious mutation of OR30 in the domestic silkworms. The sample started with “D” means domestic silkworm, “W” means wild silkworm. The sample names of wild silkworms represented the geographical sources. **b** The topology of OR30 and the location of the deleterious amino acid substitution. The topology was predicted using HMMTOP 2.1. Each circle represents an amino acid residue. The red circle represents the deleterious mutation site. TM: transmembrane helix

The ratio of the numbers of non-synonymous SNPs to the numbers of synonymous SNPs (*N*/*S*) can be used to assess the relaxation of purifying selection [43]. We counted the population-unique non-synonymous (N) and synonymous (S) SNPs for the differentially expressed olfactory genes (Table 3). The *N/S* ratio for ORs in the domestic silkworm (0.82) was significantly higher than that in the wild silkworm (0.46) (*P* < 0.01, Fisher’s exact test one-tailed). The global *d*_*N*_*/d*_*S*_ ratios and population-unique *N/S* ratios may not be enough to detect functional relaxation in the domestic silkworm. To assess the effect of a coding non-synonymous variant, we used ‘Sorting Tolerant From Intolerant’ (SIFT) algorithm [44] to predict whether population-unique non-synonymous SNP in the OR genes are tolerant or intolerant. It was indicated that one deleterious amino acid mutation was found in OR30 of the domestic population (Fig. 7a). The topology of the OR proteins was predicted using HMMTOP 2.1 (http://www.sacs.ucsf.edu/cgi-bin/hmmtop.py). The deleterious mutation of OR30 was located in the transmembrane helix 6 (TM6). Compared with *B. mandarina*, functional redundancy of olfaction might have the effect of increasing population-unique *N/S* ratio and accumulating deleterious polymorphisms in the domestic silkworm.

## Discussion

Due to long-term exposure to different environments, the domestic silkworm and its wild relative *B. mandarina* showed different olfactory sensitivity to the plant odorants [1]. Comparative study of antennal transcriptomes in indoor and wild species may help us understand the potential mechanisms for impairing olfactory sensitivity under domestication. In the adult antennal transcriptomes of the domestic and wild silkworms, 22,767 unigenes were assembled, in which over 57.45% of the unigenes were expressed (FPKM ≥1) (Additional file 4: Table S4). In insects, olfactory genes and odorant degrading enzymes mediate the olfactory response [2, 41]. In this study, some novel olfactory-related genes were characterized, especially, ORs and IRs (Table 1). We found that more than half of the olfactory-related genes were expressed in the antennae of the domestic and wild silkworms (Table 1, Additional file 4: Table S4). These expressed olfactory-related genes may be involved in processes that are directly or indirectly connected to sensory perception.

During sexual reproduction, female-produced sex pheromones guide flying males to their mates. Some function-specific odorant receptors and binding proteins provide vital links between sex pheromones and sexual behavior. In the silkworm, *BmOR1*, *BmOR3* and *BmPBP1* are the most important sex pheromone receptors and binding proteins [7, 8, 45], which showed male-biased or male-abundant expression patterns (Additional file 11: Table S9). Interestingly, the three genes have similar expression levels and no significant differences in males between the domestic and wild silkworms (Additional file 11: Table S9). Previous studies indicated that the sex pheromones bombykol and bombykal evoked odorant-specific electroantennography (EAG) amplitudes were comparable between the domestic and wild silkworm males [1]. Our result is in accord with the neurophysiological consequences, suggesting that pheromone communication is equally important for indoor and wild species. This may be because sexual behavior might have been under strong stabilizing selection to maintain population reproduction both in domestic species, *B. mori*, and wild silkworm [1].

As a part of olfaction response, odorant-degrading enzymes are crucial to inactivation of stimulus molecules to avoid the continuous stimulation of the receptors [2, 41, 46]. For instance, the antennal GSTs can modify trans-2-hexenal, a plant-derived green leaf aldehyde known to stimulate the olfactory system of *Manduca sexta* [47]. It was indicated that an antennal COE, *esterase-6*, showed a wide range of functions in degrading for many bioactive food esters in *D. melanogaster* [6, 48]. Previous studies have found that a number of putative ODEs were expressed in the antennae of the *D. melanogaster* [46], *Spodoptera littoralis* [49], *Holotrichia parallela* [50], and etc. In this study, 127 putative ODEs were expressed in the antennae of the domestic and wild silkworms (Additional file 10: Table S4). Furthermore, we identified 19 differentially expressed ODEs in D_M vs. W_M and D_F vs. W_F, of which 14 ODEs were down-regulated in the domestic silkworm (Fig. 5). Although the function of those differentially expressed ODEs has not been validated in the silkworm yet, they may have a similar function in degrading plant odorants with other species. Due to down-regulation of a great deal of the differentially expressed ODEs, it might affect the inactivation efficiency of plant stimulus molecules, and decrease the perception in domestic silkmoth [1]. Compared with the domestic silkworm, the wild silkmoth would encounter more complex volatiles in the field, such as non-host plant odorants. The up-regulation of 14 out of 19 differentially expressed ODEs may play roles in inactivate extra volatiles and help wild silkmoth maintain its olfactory sensitivities for locating mates and an appropriate oviposition site. In addition, insects are often exposed to direct and residual contacts with toxic volatiles, especially insecticides, which may impair olfactory performances involved in scent recognition and neural treatment [41, 51]. Odorant-degrading enzymes may play important roles in detoxification of volatile xenobiotics [2, 41, 52]. Relatively, wild silkmoth would encounter much more toxic volatiles than indoor *B. mori*. It was suggested that the up-regulated ODEs might help wild silkworm detoxify toxic odorants to keep sensory processing sensitivity too.

In order to retain olfactory sensitivity, a proper expression level of olfactory genes, such as ORs and OBPs, is a key mechanism. In *Dendroctonus armandi*, RNA interference (RNAi) was used to reduce the expression of an odorant receptor [53]. It was found that antennae of RNAi-treated *D. armandi* showed significantly lower electrophysiological responses to 11 major volatiles of its host [53]. Similar studies for OBPs were conducted in *D. melanogaster* and *Culex quinquefasciatus* [13, 14]. Because the ecological niche of *B. mori* has changed greatly since domestication from *B. mandarina*. *B. mori* has become entirely dependent on humans for survival and does not need to seek oviposition sites and food in a complex environment. *B. mandarina* lives in an outdoor environment and need sensitive olfaction for mating and finding oviposition sites [1]. In this study, the pupae of the domestic and wild silkworms were put in an identical indoor chamber until dissection of adult antennae. The differential expressions of the candidate genes might be caused by the divergence of the genomic background during long-term adaptation. Through the comparisons of the same sex between the domestic and wild silkworms, 30 differentially expressed olfactory genes were identified (Fig. 5). In addition, seven CSPs (*CSP3*, *CSP8*, *CSP10*, *CSP11*, CSP12, *CSP18*, and *CSP19*) were also identified as DEGs (Additional file 8: Table S7). Unexceptionally, all of them were up-regulated the domestic silkworm. Based on the microarray data of the tissues and developmental stages in the silkworms [54, 55], *CSP3*, *CSP8*, *CSP11*, *CSP12* showed high expressions in various tissues and developmental stages (Additional file 12: Figure S3). We suspect that the up-regulated expressions of CSPs were not the reason for impairing olfactory sensitivity in the domestic silkworm, while its roles need to be explored in the future. For the 30 differentially expressed olfactory genes, 19 were down-regulated in the domestic silkworm, and almost all of them were ORs and OBPs (Fig. 5). In the silkworm, some of the ORs have been characterized for ligand responsiveness [9, 10]. The previous study indicated that BmOR56 showed a high sensitivity to *cis*-jasmone, which is a potent attractant in mulberry leaves for silkworm [9]. In the domestic silkworm, it might be no need for highly sensitive olfaction to plant volatiles, which may result in down-regulation of *BmOR56* and the other olfactory genes. However, the high background expression levels of the olfactory genes should be beneficial for wild silkworm to maintain a high perception of plant volatiles.

Except for expression level of the olfactory genes, sequence polymorphisms would also contribute to olfactory sensitivity [16, 18]. Nineteen olfactory genes were down-regulated in the domestic silkworm, of which 18 were ORs and OBPs (Fig. 5). We focused on the 17 functional OBPs and ORs excluded the pseudogene *OR48* [9]. Molecular population parameters and functional constraints were examined in the domestic and wild populations. In coding regions, the nucleotide diversities of the 17 olfactory genes showed lower nucleotide diversity in the domestic silkworms than wild silkworms (Table 2). This reduction in nucleotide diversity is likely due to inbreeding or the bottleneck experienced by domesticated strains [20]. Neutral test on the olfactory genes displayed negative values appeared in the tested wild population (Table 2), which may be caused by positive selection or negative selection [18]. Thus, natural selection may play important roles in maintaining olfactory sensitivity for host plant volatiles in the field.

Due to inbreeding and bottleneck effect, genetic diversities (π) of the 17 differentially expressed olfactory genes were lower in the domestic silkworm (Table 2). However, the global *d*_*N*_*/d*_*S*_ ratios of the differentially expressed olfactory genes were comparable between the domestic and wild silkworms (Fig. 6). Thus, the relaxed purifying selection may drive an increase of non-synonymous evolutionary rate in the domestic silkworms. To refine this result, we found that the domestic silkworm has a higher population-unique non-synonymous/synonymous (*N/S*) ratio for the ORs than the wild silkworms (Table 3). Furthermore, we focused on the population-unique non-synonymous sites of the 13 OR genes. The potentially tolerated/intolerant mutations were predicted by SIFT tool [44]. In the domestic population, one deleterious mutation was found in OR30, which was located in transmembrane helix 6 (TM6) (Fig. 7). In insects, the previous studies indicated that TM5–7 region of ORs was formed as a central part of the ion-conducting channel, in which many mutations have general deleterious effects on ion channel function and ion selectivity [56, 57]. For instance, mutation of Y464 (in TM7) would affect K^+^ selectivity in *Bombyx mori* ORCO [57]. Except for the silkworms, accumulation of deleterious and intolerant mutations in olfactory genes was also found in domestic dogs [18]. In future, the odorant ligands of OR30 and effects of the deleterious mutations on olfaction sensitivity need to be validated. It would help us understand whether the accumulation of deleterious mutations driven by relaxed selection is one of the mechanisms for impairing olfactory sensitivity in domestic silkmoth.

## Conclusions

Comparative analysis of the antennal transcriptomes was conducted in the domestic and wild silkworms. The differentially expressed genes related to odor perception were identified, including 30 olfactory genes and 19 ODEs, most of which were down-regulated in the domestic silkworm. Furthermore, the down-regulated ORs showed a higher population-unique *N/S* ratio in the domestic populations than that in the wild silkworms. Especially, one deleterious mutation was found in OR30 in the domestic populations. It was indicated that down-regulation and functional relaxation of the olfactory-related genes might impair the olfactory sensitivity to environmental odorants in domestic silkworms. This study provides insights into the molecular mechanisms of olfactory impairment in the domestic silkworms. More importantly, it may help us understand the potential mechanisms for retaining olfactory sensitivity in wild conditions.

## Additional files

Additional file 1: **Table S1.** Primer sequences used for the qPCR validation experiment. (DOCX 15 kb)

Additional file 2: **Table S2.** Statistics of RNA-Seq data after quality control. R1 and R2 at the end of the sample name represent repeat 1 and 2, respectively. Gb: Giga base; Q20: percentage of bases with a Phred value of at least 20. Q30: percentage of bases with a Phred value of at least 30. (DOCX 16 kb)

Additional file 3: **Table S3.** Summary of the clean reads mapped to the silkworm reference genome. (XLSX 11 kb)

Additional file 4: **Table S4.** Expression levels of all genes across the four antennal samples. The accession number with “BGIBMGA” was from SilkDB (http://www.silkdb.org/silkdb/), and the others from GenBank in NCBI. A BLAST search was conducted using the all unique transcripts as query sequences against the protein non-redundant (nr) database in NCBI. The best BLAST hits and *E*-values are listed. (XLSX 2319 kb)

Additional file 5: **Table S5.** Distribution of gene expressions in adult antennae of the domestic and wild silkworms. FPKM: Fragments Per Kilobase of transcript per Million fragments mapped. The female (W_F) and male (W_M) of the wild silkworm, female (D_F) and male (D_M) of the domestic silkworm were showed. (DOCX 15 kb)

Additional file 6: **Figure S1.** Annotation of KEGG pathway for all the 22,767 unigenes. All the pathways were included in six categories: Metabolism, Genetic Information Processing, Environmental Information Processing, Cellular Processes, Organismal Systems, and Human Diseases. (PDF 232 kb)

Additional file 7: **Table S6.** The sequences of the olfactory-related novel genes. (DOCX 28 kb)

Additional file 8: **Table S7.** Differentially expressed genes between any two antennal samples. The *P*-values were adjusted for multiple testing using the Benjamini-Hochberg method. An adjusted *P*-value (padj) of 0.05 and fold-change of 3 was set as the threshold for significant differential expression. (XLSX 559 kb)

Additional file 9: **Figure S2.** Scatterplot of enriched GO terms related to olfaction for all the differentially expressed genes. (PDF 156 kb)

Additional file 10: **Table S8.** Gene Ontology enrichment analysis of the DEGs. D_Down and D_Up mean down- and up-regulated in the domestic silkworms, respectively. (XLSX 351 kb)

Additional file 11: **Table S9.** The sex-biased genes in the domestic and wild silkworms. The putative functions were from annotations through BLAST against nr database in NCBI, and the detailed information of BLAST best hits was in Additional file 4: Table S4, Supporting information. (DOCX 16 kb)

Additional file 12: **Figure S3.** Expression profiles of the differentially expressed CSPs in the tissues and developmental stages of the silkworm. The microarrays of tissues and development stages in the silkworm were retrieved from the previous studies [54, 55]. The expression signals were used to plot. When the expression signal was higher than 400, it was considered that this gene has expression evidence. (PDF 347 kb)

## Abbreviations

DEGs: Differentially expressed genes
FPKM: Fragments Per Kilobase of transcript per Million fragments mapped
GO: Gene ontology
KEGG: Kyoto Encyclopedia of Genes and Genomes

## References

[1] Bisch-Knaden S, Daimon T, Shimada T, Hansson BS, Sachse S (2014). Anatomical and functional analysis of domestication effects on the olfactory system of the silkmoth *Bombyx mori*. P Roy Soc B-Biol Sci..

[2] Leal WS (2013). Odorant reception in insects: roles of receptors, binding proteins, and degrading enzymes. Annu Rev Entomol..

[3] Fleischer J, Pregitzer P, Breer H, Krieger J (2018). Access to the odor world: olfactory receptors and their role for signal transduction in insects. Cell Mol Life Sci.

[4] Zhou JJ (2010). Odorant-binding proteins in insects. Vitam Horm.

[5] Ishida Y, Leal WS (2005). Rapid inactivation of a moth pheromone. Proc Natl Acad Sci U S A.

[6] Chertemps T, Younus F, Steiner C, Durand N, Coppin CW, Pandey G (2015). An antennal carboxylesterase from *Drosophila melanogaster*, esterase 6, is a candidate odorant-degrading enzyme toward food odorants. Front Physiol.

[7] Nakagawa T, Sakurai T, Nishioka T, Touhara K (2005). Insect sex-pheromone signals mediated by specific combinations of olfactory receptors. Science.

[8] Sakurai T, Nakagawa T, Mitsuno H, Mori H, Endo Y, Tanoue S (2004). Identification and functional characterization of a sex pheromone receptor in the silkmoth *Bombyx mori*. Proc Natl Acad Sci U S A.

[9] Tanaka K, Uda Y, Ono Y, Nakagawa T, Suwa M, Yamaoka R (2009). Highly selective tuning of a silkworm olfactory receptor to a key mulberry leaf volatile. Curr Biol.

[10] Anderson AR, Wanner KW, Trowell SC, Warr CG, Jaquin-Joly E, Zagatti P (2009). Molecular basis of female-specific odorant responses in *Bombyx mori*. Insect Biochem Mol Biol.

[11] Sun W, Yu H, Shen Y, Banno Y, Xiang Z, Zhang Z (2012). Phylogeny and evolutionary history of the silkworm. Sci China Life Sci.

[12] Gadenne C, Barrozo RB, Anton S (2016). Plasticity in insect olfaction: to smell or not to smell?. Annu Rev Entomol.

[13] Swarup S, Williams TI, Anholt RRH (2011). Functional dissection of odorant binding protein genes in *Drosophila melanogaster*. Genes Brain Behav.

[14] Pelletier J, Guidolin A, Syed Z, Cornel AJ, Leal WS (2010). Knockdown of a mosquito odorant-binding protein involved in the sensitive detection of oviposition attractants. J Chem Ecol.

[15] Liu Q, Liu W, Zeng B, Wang G, Hao D, Huang Y (2017). Deletion of the *Bombyx mori* odorant receptor co-receptor (BmOrco) impairs olfactory sensitivity in silkworms. Insect Biochem Mol Biol.

[16] Wang P, Lyman RF, Mackay TFC, Anholt RRH (2010). Natural variation in odorant recognition among odorant-binding proteins in *Drosophila melanogaster*. Genetics..

[17] Wang P, Lyman RF, Shabalina SA, Mackay TFC, Anholt RRH (2007). Association of polymorphisms in odorant-binding protein genes with variation in olfactory response to benzaldehyde in *Drosophila*. Genetics..

[18] Chen R, Irwin DM, Zhang YP (2012). Differences in selection drive olfactory receptor genes in different directions in dogs and wolf. Mol Biol Evol..

[19] Patel RK, Jain M (2012). NGS QC toolkit: a toolkit for quality control of next generation sequencing data. PloS one.

[20] Xia Q, Guo Y, Zhang Z, Li D, Xuan Z, Li Z (2009). Complete resequencing of 40 genomes reveals domestication events and genes in silkworm (*Bombyx*). Science.

[21] Fang SM, Hu BL, Zhou QZ, Yu QY, Zhang Z (2015). Comparative analysis of the silk gland transcriptomes between the domestic and wild silkworms. BMC Genomics.

[22] Langmead B, Trapnell C, Pop M, Salzberg SL (2009). Ultrafast and memory-efficient alignment of short DNA sequences to the human genome. Genome Biol.

[23] Kim D, Pertea G, Trapnell C, Pimentel H, Kelley R, Salzberg SL (2013). TopHat2: accurate alignment of transcriptomes in the presence of insertions, deletions and gene fusions. Genome Biol.

[24] Roberts A, Pimentel H, Trapnell C, Pachter L (2011). Identification of novel transcripts in annotated genomes using RNA-Seq. Bioinformatics.

[25] Anders S, Pyl PT, Huber W (2015). HTSeq--a Python framework to work with high-throughput sequencing data. Bioinformatics.

[26] Anders S, Huber W (2010). Differential expression analysis for sequence count data. Genome Biol.

[27] Conesa A, Gotz S, Garcia-Gomez JM, Terol J, Talon M, Robles M (2005). Blast2GO: a universal tool for annotation, visualization and analysis in functional genomics research. Bioinformatics.

[28] Edgar RC (2004). MUSCLE: multiple sequence alignment with high accuracy and high throughput. Nucleic Acids Res.

[29] Darriba D, Taboada GL, Doallo R, Posada D (2011). ProtTest 3: fast selection of best-fit models of protein evolution. Bioinformatics.

[30] Stamatakis A (2014). RAxML version 8: a tool for phylogenetic analysis and post-analysis of large phylogenies. Bioinformatics.

[31] Kumar S, Stecher G, Li M, Knyaz C, Tamura K (2018). MEGA X: molecular evolutionary genetics analysis across computing platforms. Mol Biol Evol.

[32] Croset V, Rytz R, Cummins SF, Budd A, Brawand D, Kaessmann H (2010). Ancient protostome origin of chemosensory ionotropic glutamate receptors and the evolution of insect taste and olfaction. PLoS Genet.

[33] DePristo MA, Banks E, Poplin R, Garimella KV, Maguire JR, Hartl C (2011). A framework for variation discovery and genotyping using next-generation DNA sequencing data. Nature Genet.

[34] Li H, Handsaker B, Wysoker A, Fennell T, Ruan J, Homer N (2009). The sequence alignment/map format and SAMtools. Bioinformatics.

[35] Garrison E, Marth G (2012). Haplotype-based variant detection from short-read sequencing. arXiv preprint arXiv:12073907 [q-bioGN].

[36] Librado P, Rozas J (2009). DnaSP v5: a software for comprehensive analysis of DNA polymorphism data. Bioinformatics.

[37] Yang Z (2007). PAML 4: phylogenetic analysis by maximum likelihood. Mol Biol Evol.

[38] Yu QY, Fang SM, Zhang Z, Jiggins CD (2016). The transcriptome response of *Heliconius melpomene* larvae to a novel host plant. Mol Ecol.

[39] Vogt RG, Grosse-Wilde E, Zhou JJ (2015). The lepidoptera odorant binding protein gene family: gene gain and loss within the GOBP/PBP complex of moths and butterflies. Insect Biochem Mol Biol.

[40] Koenig C, Hirsh A, Bucks S, Klinner C, Vogel H, Shukla A (2015). A reference gene set for chemosensory receptor genes of *Manduca sexta*. Insect Biochem Mol Biol.

[41] Engsontia P, Sangket U, Chotigeat W, Satasook C (2014). Molecular evolution of the odorant and gustatory receptor genes in lepidopteran insects: implications for their adaptation and speciation. J Mol Evol.

[42] Vogt RG, Miller NE, Litvack R, Fandino RA, Sparks J, Staples J (2009). The insect SNMP gene family. Insect Biochem Mol Biol.

[43] Kulmuni J, Havukainen H (2013). Insights into the evolution of the CSP gene family through the integration of evolutionary analysis and comparative protein modeling. PloS one..

[44] Yu Q, Lu C, Li B, Fang S, Zuo W, Dai F (2008). Identification, genomic organization and expression pattern of glutathione S-transferase in the silkworm, *Bombyx mori*. Insect Biochem Mol Biol..

[45] Ahn SJ, Vogel H, Heckel DG (2012). Comparative analysis of the UDP-glycosyltransferase multigene family in insects. Insect Biochem Mol Biol.

[46] Yu QY, Lu C, Li WL, Xiang ZH, Zhang Z (2009). Annotation and expression of carboxylesterases in the silkworm, *Bombyx mori*. BMC Genomics.

[47] Ai J, Zhu Y, Duan J, Yu Q, Zhang G, Wan F, Xiang ZH (2011). Genome-wide analysis of cytochrome P450 monooxygenase genes in the silkworm, *Bombyx mori*. Gene.

[48] Pelletier J, Bozzolan F, Solvar M, Francois MC, Jacquin-Joly E, Maibeche-Coisne M (2007). Identification of candidate aldehyde oxidases from the silkworm *Bombyx mori* potentially involved in antennal pheromone degradation. Gene.

[49] Livak KJ, Schmittgen TD (2001). Analysis of relative gene expression data using real-time quantitative PCR and the 2(T)(-Delta Delta C) method. Methods.

[50] Benton R, Vannice KS, Gomez-Diaz C, Vosshall LB (2009). Variant ionotropic glutamate receptors as chemosensory receptors in *Drosophila*. Cell.

[51] Vogt RG, Gilbert LI, Iatrou K, Gill SS (2005). Molecular basis of pheromone detection in insects. Comprehensive insect physiology, biochemistry, pharmacology and molecular biology.

[52] Wang MS, Zhang RW, Su LY, Li Y, Peng MS, Liu HQ (2016). Positive selection rather than relaxation of functional constraint drives the evolution of vision during chicken domestication. Cell Res.

[53] Kumar P, Henikoff S, Ng PC (2009). Predicting the effects of coding non-synonymous variants on protein function using the SIFT algorithm. Nature Protoc.

[54] Krieger J, von Nickisch-Rosenegk E, Mameli M, Pelosi P, Breer H (1996). Binding proteins from the antennae of *Bombyx mori*. Insect Biochem Mol Biol.

[55] Younus F, Chertemps T, Pearce SL, Pandey G, Bozzolan F, Coppin CW (2014). Identification of candidate odorant degrading gene/enzyme systems in the antennal transcriptome of *Drosophila melanogaster*. Insect Biochem Mol Biol.

[56] Rogers ME, Jani MK, Vogt RG (1999). An olfactory-specific glutathione-S-transferase in the sphinx moth *Manduca sexta*. J Exp Biol.

[57] Younus F, Fraser NJ, Coppin CW, Liu JW, Correy GJ, Chertemps T (2017). Molecular basis for the behavioral effects of the odorant degrading enzyme Esterase 6 in *Drosophila*. Sci Rep.

[58] Belzunces LP, Tchamitchian S, Brunet J (2012). Neural effects of insecticides in the honey bee. Apidologie.

[59] Durand N, Carot-Sans G, Chertemps T, Montagne N, Jacquin-Joly E, Debernard S (2010). A diversity of putative carboxylesterases are expressed in the antennae of the noctuid moth *Spodoptera littoralis*. Insect Mol Biol.

[60] Wang S, Liu Y, Zhou JJ, Yi JK, Pan Y, Wang J (2018). Identification and tissue expression profiling of candidate UDP-glycosyltransferase genes expressed in *Holotrichia parallela* motschulsky antennae. Bull Entomol Res.

[61] Li X, Schuler MA, Berenbaum MR (2007). Molecular mechanisms of metabolic resistance to synthetic and natural xenobiotics. Annu Rev Entomol.

[62] Zhang R, Gao G, Chen H (2016). Silencing of the olfactory co-receptor gene in *Dendroctonus armandi* leads to EAG response declining to major host volatiles. Sci Rep.

[63] Xia Q, Cheng D, Duan J, Wang G, Cheng T, Zha X (2007). Microarray-based gene expression profiles in multiple tissues of the domesticated silkworm, *Bombyx mori*. Genome Biol.

[64] Wang GH, Jiang L, Zhu L, Cheng TC, Niu WH, Yan YF (2013). Characterization of Argonaute family members in the silkworm, *Bombyx mori*. Insect Sci.

[65] Hopf TA, Morinaga S, Ihara S, Touhara K, Marks DS, Benton R (2015). Amino acid coevolution reveals three-dimensional structure and functional domains of insect odorant receptors. Nat Commun.

[66] Nakagawa T, Pellegrino M, Sato K, Vosshall LB, Touhara K (2012). Amino acid residues contributing to function of the heteromeric insect olfactory receptor complex. PloS one..

