# Supplementary material for: Evidence of peripheral olfactory impairment in the domestic silkworms: insight from the comparative transcriptome and population genetics
Source: BMC Genomics. 2018 Nov 1;19:788. doi: 10.1186/s12864-018-5172-1 (PMC6211594; doi:10.1186/s12864-018-5172-1)
Supplement: Supplementary file 1 — Table S1. Primer sequences used for the qPCR validation experiment. (DOCX 15 kb) [file 12864_2018_5172_MOESM1_ESM.docx]

**Table S1 Primer sequences used for the qPCR validation experiment.**

| **Gene name** | **Forward primer (5'-3')** | **Reverse primer (5'-3')** | **Length of amplificon (bp)** | **Annealing temperature (°C)** |
| --- | --- | --- | --- | --- |
| *BmCSP11* | GTATCGGTCGTTGTTTGCC | GATGACGCTGTTTCGGGTT | 217 | 53 |
| *BmCSP12* | TGAAGGCCTTTATTGGATGC | GCTTTCCTTGGGGATCGTAT | 213 | 53 |
| *BmCSP18* | CTGCTGCCCGAAGTGATAG | CGTTTGCCAGTCTGTTGTT | 230 | 50 |
| *BmCSP19* | AGAACCCGAGGCTATTGAA | TGAGCGTCTCAGGGAAGTA | 192 | 50 |
| *BmCSP3* | CTGTTGGCCGCCTGTCTTG | GCACTTCCCGCAGTTGGTT | 189 | 55 |
| *BmCSP8* | AATTCCCTCATCGCATTCTG | TCTGGGCTTCGGTACATTTC | 244 | 55 |
| *BmGOBP2* | TTCTCTGATGGACGATGACG | TCTGGTGCTATGCCCTCTTT | 214 | 55 |
| *BmOBP10* | ACCTGCAACAGGAGAAAACG | GCAACCTTGTCCCTTGTCAT | 214 | 55 |
| *BmOBP11* | TGCCTCTAATAGCGGAATG | AAAGACCACTTGCGTTGAT | 150 | 50 |
| *BmOBP15* | CGAAACAGCAAATAAAAAACTCT | ATATCGACCTGCTTCACCA | 211 | 50 |
| *BmOBP28* | CAGGAGCGAATCCGAACTT | TAGCGCAACACGAACTGGAG | 284 | 50 |
| *BmOR39* | TTTACGATTGTCGGTGGGA | AAGCGTGGTGTAGGCAGAG | 170 | 53 |
| *BmPBP2* | TCCCGAGATGTGATGACTAA | TGAGGTCCACTAGCTGTTTT | 280 | 50 |
| *RpL3* | CGGTGTTGTTGGATACATTGAG | GCTCATCCTGCCATTTCTTACT | 161 | 55 |
