# Supplementary material for: Evidence of peripheral olfactory impairment in the domestic silkworms: insight from the comparative transcriptome and population genetics
Source: BMC Genomics. 2018 Nov 1;19:788. doi: 10.1186/s12864-018-5172-1 (PMC6211594; doi:10.1186/s12864-018-5172-1)
Supplement: Supplementary file 2 — Table S2. Statistics of RNA-Seq data after quality control. R1 and R2 at the end of the sample name represent repeat 1 and 2, respectively. Gb: Giga base; Q20: percentage of bases with a Phred value of at least 20. Q30: percentage of bases with a Phred value of at least 30. (DOCX 16 kb) [file 12864_2018_5172_MOESM2_ESM.docx]

**Table S2** **Statistics of RNA-Seq data after quality control**

| **Sample name** | **Raw reads** | **Clean reads** | **Clean**  **data (GB)** | **Error**  **rate (%)** | **Q20 (%)** | **Q30 (%)** | **GC content (%)** |
| --- | --- | --- | --- | --- | --- | --- | --- |
| D_M_R1 | 26,783,016 | 26,311,264 | 6.58 | 0.04 | 95.22 | 91.06 | 45.41 |
| D_M_R2 | 31,889,503 | 31,741,469 | 8.92 | 0.03 | 96.11 | 90.83 | 46.79 |
| D_F_R1 | 24,779,010 | 24,588,382 | 6.15 | 0.03 | 96.38 | 92.88 | 45.20 |
| D_F_R2 | 27,486,249 | 25,755,170 | 7.50 | 0.06 | 94.48 | 86.25 | 46.56 |
| W_M_R1 | 29,692,545 | 28,849,329 | 7.21 | 0.04 | 95.19 | 90.97 | 44.53 |
| W_M_R2 | 29,899,925 | 29,745,830 | 8.10 | 0.04 | 94.59 | 87.72 | 47.10 |
| W_F_R1 | 33,541,462 | 32,912,105 | 8.23 | 0.04 | 95.36 | 91.26 | 44.55 |
| W_F_R2 | 31,718,235 | 31,525,177 | 8.51 | 0.04 | 94.28 | 87.30 | 43.90 |

R1 and R2 at the end of the sample name represent repeat 1 and 2, respectively. Gb: Giga base; Q20: percentage of bases with a Phred value of at least 20. Q30: percentage of bases with a Phred value of at least 30.
