# Supplementary material for: Evidence of peripheral olfactory impairment in the domestic silkworms: insight from the comparative transcriptome and population genetics
Source: BMC Genomics. 2018 Nov 1;19:788. doi: 10.1186/s12864-018-5172-1 (PMC6211594; doi:10.1186/s12864-018-5172-1)
Supplement: Supplementary file 5 — Table S5. Distribution of gene expressions in adult antennae of the domestic and wild silkworms. FPKM: Fragments Per Kilobase of transcript per Million fragments mapped. The female (W_F) and male (W_M) of the wild silkworm, female (D_F) and male (D_M) of the domestic silkworm were showed. (DOCX 15 kb) [file 12864_2018_5172_MOESM5_ESM.docx]

**Table S5 Distribution of gene expressions in adult antennae of the domestic and wild silkworms**

| **FPKM interval** | **D_M (**%**)** | **D_F (**%**)** | **W_M (**%**)** | **W_F (**%**)** |
| --- | --- | --- | --- | --- |
| > 1000 | 74 (0.33) | 69 (0.30) | 61 (0.27) | 66 (0.29) |
| 100-1000 | 578 (2.54) | 580 (2.55) | 584 (2.57) | 595 (2.61) |
| 10-100 | 3844 (16.88) | 4309 (18.93) | 3815 (16.76) | 4201 (18.45) |
| 1-10 | 8584 (37.70) | 9064 (39.81) | 8782 (38.57) | 9101 (39.98) |
| 0-1 | 9687 (42.55) | 8745 (38.41) | 9525 (41.84) | 8804 (38.67) |

FPKM: Fragments Per Kilobase of transcript per Million fragments mapped. The female (W_F) and male (W_M) of the wild silkworm, female (D_F) and male (D_M) of the domestic silkworm were showed.
