# Supplementary figures and images for: Evidence of peripheral olfactory impairment in the domestic silkworms: insight from the comparative transcriptome and population genetics
Source: BMC Genomics. 2018 Nov 1;19:788. doi: 10.1186/s12864-018-5172-1 (PMC6211594; doi:10.1186/s12864-018-5172-1)

# KEGG pathway annotation

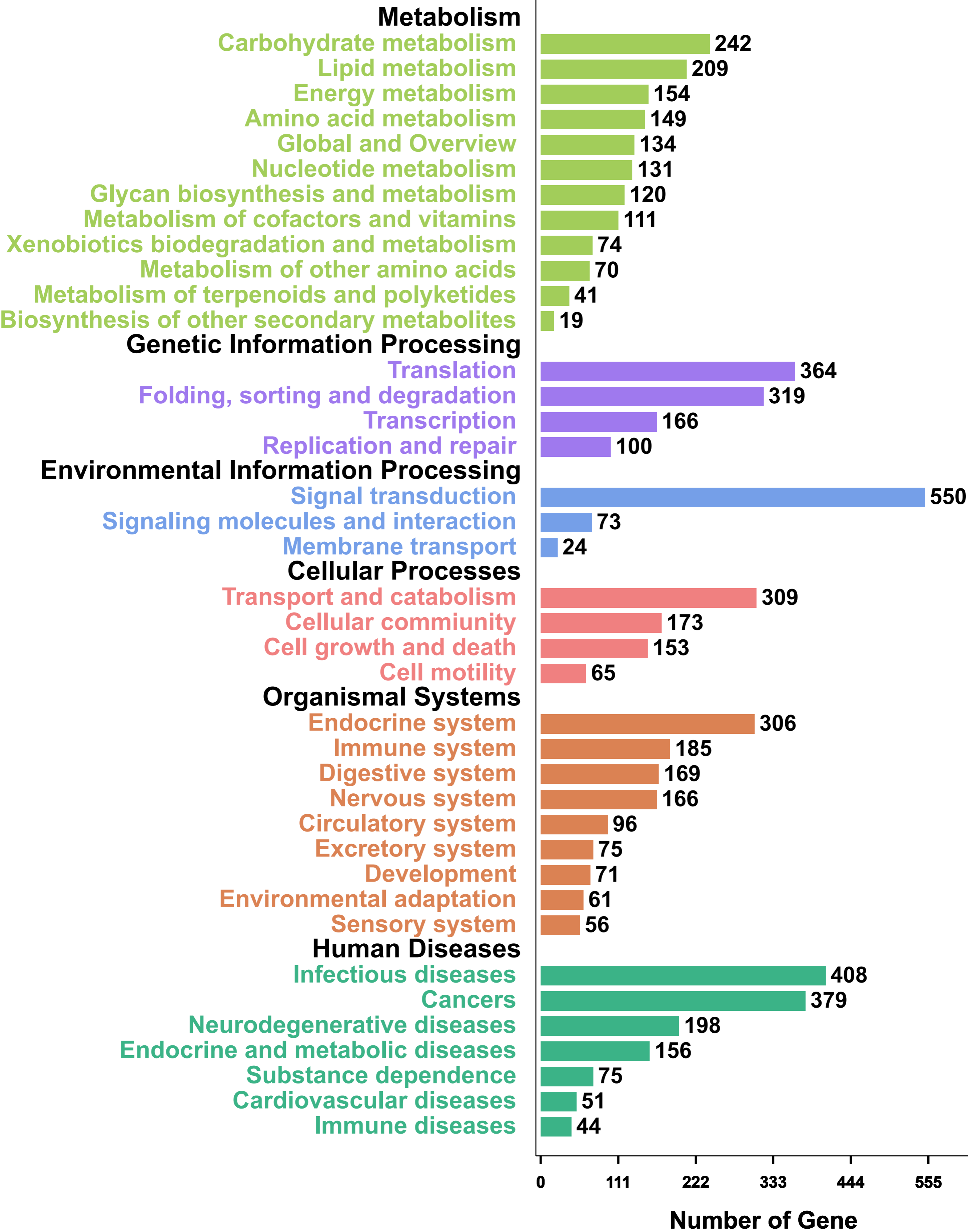

Supplement: Supplementary file 6 — Figure S1. Annotation of KEGG pathway for all the 22,767 unigenes. All the pathways were included in six categories: Metabolism, Genetic Information Processing, Environmental Information Processing, Cellular Processes, Organismal Systems, and Human Diseases. (PDF 232 kb) [file 12864_2018_5172_MOESM6_ESM.pdf]

Statistics of GO Enrichment

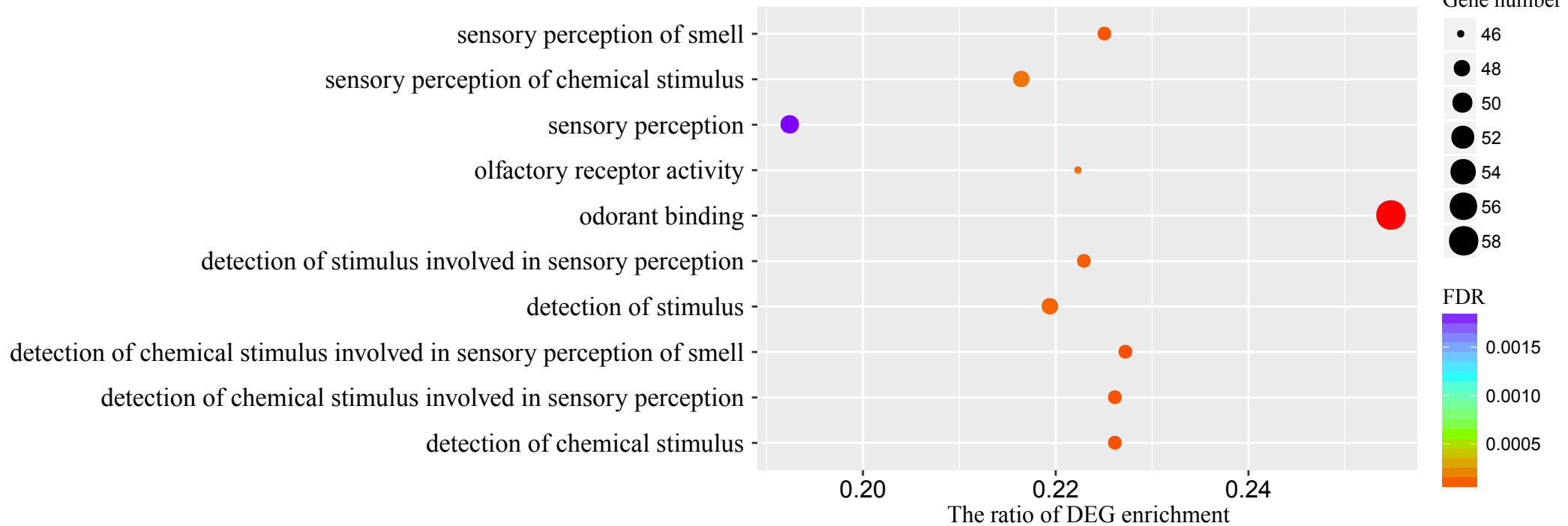

Supplement: Supplementary file 9 — Figure S2. Scatterplot of enriched GO terms related to olfaction for all the differentially expressed genes. (PDF 156 kb) [file 12864_2018_5172_MOESM9_ESM.pdf]

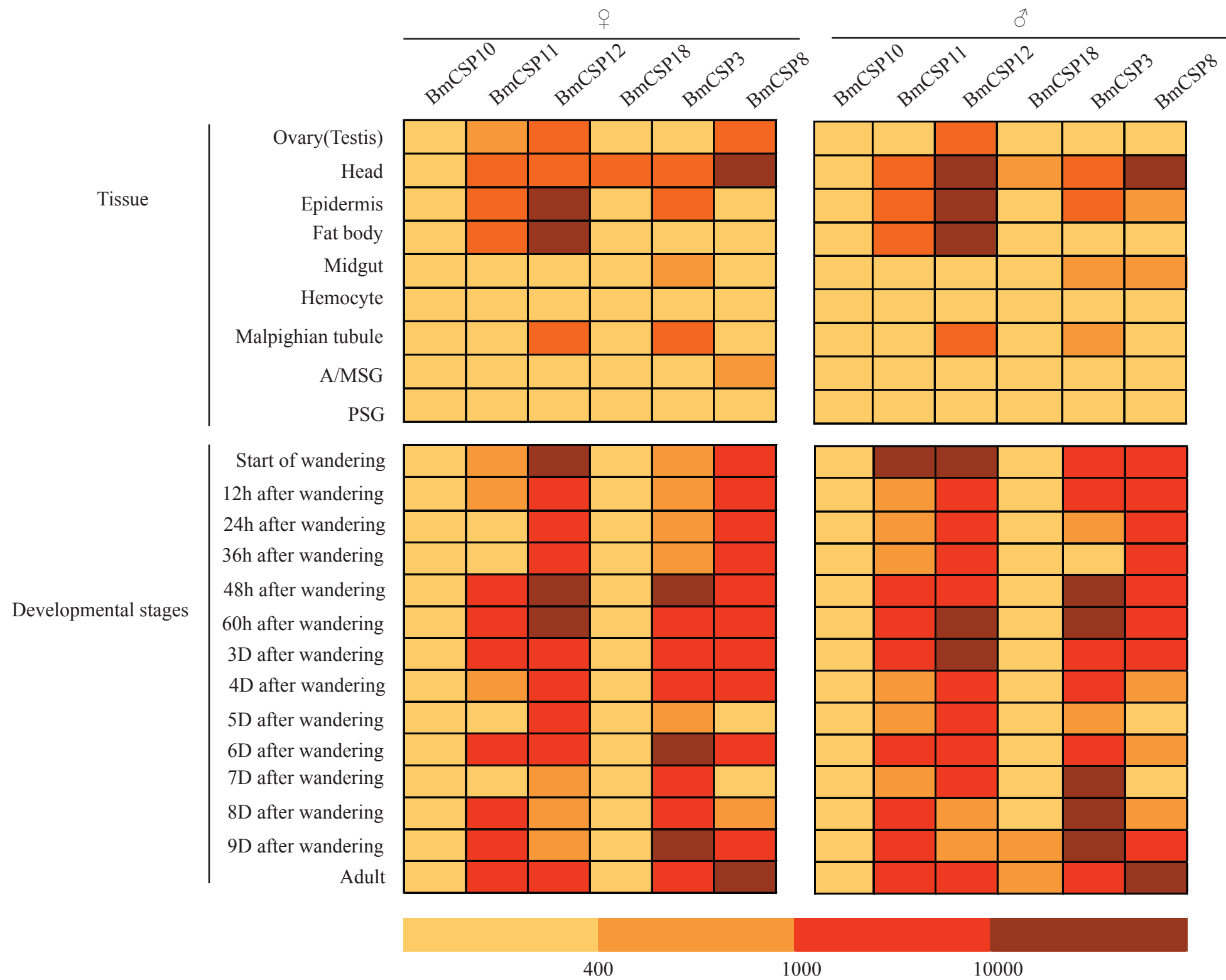

Supplement: Supplementary file 12 — Figure S3. Expression profiles of the differentially expressed CSPs in the tissues and developmental stages of the silkworm. The microarrays of tissues and development stages in the silkworm were retrieved from the previous studies [54, 55]. The expression signals were used to plot. When the expression signal was higher than 400, it was considered that this gene has expression evidence. (PDF 347 kb) [file 12864_2018_5172_MOESM12_ESM.pdf]
