# Supplementary material for: Evidence of peripheral olfactory impairment in the domestic silkworms: insight from the comparative transcriptome and population genetics
Source: BMC Genomics. 2018 Nov 1;19:788. doi: 10.1186/s12864-018-5172-1 (PMC6211594; doi:10.1186/s12864-018-5172-1)
Supplement: Supplementary file 7 — Table S6. The sequences of the olfactory-related novel genes. (DOCX 28 kb) [file 12864_2018_5172_MOESM7_ESM.docx]

**The novel olfactory-related genes in the silkworm**

>BmOR74

Atgatactcactcaacaagaaaaggtcatgatggtgtccgaaaaaatacgcatgtttgatagatcttccgtgtatggaggtctaatagaaacaatatctaatttcctaacagttttgaacttgattttaattgtgtttctgatagcacttggaggacaactattgtgtgacacgagctctaacatttctgacgccgcttacaacagcctctggtatgaaggcgacctcaaacttagaaaactaatgttgtttatcatcactcgatcacaaaatccgtgctatttatctgcattgggattttcaaatatgactctacgatcattttcaaagattatgagcacagcatggacgtatctgtcgcttcttattcaagtttatgaaggaaattaa

>BmOR74

MILTQQEKVMMVSEKIRMFDRSSVYGGLIETISNFLTVLNLILIVFLIALGGQLLCDTSSNISDAAYNSLWYEGDLKLRKLMLFIITRSQNPCYLSALGFSNMTLRSFSKIMSTAWTYLSLLIQVYEGN

>BmOR75

ATGGAAGCCTTAAAAGATTATCCTGTTGATTTTGCGAGATCCTTCAAGGCCAGTTTGGACTTCCTCTCCTGGAGTAACATAAAGTTTTTCGCCGAGGATCAAACATTCATTCAACGATATTGGCGATTGATTATCACAACACCTTGCGTTGTTTTGATGTATGTTACAGCAATTCTGCATATATGCAAGTTGTTGTACGAGGAGGAGATATTTGTAATTGCATACCTTATGCCCCCCGTTTTGGTTGCGACTCAAGCGATTCTGAAAGCGTTCGTTTTGGTGCCGAACACTTCACAAGTGTCACACATAATGCGGGAACTTGGGAGCTTGTGGACGAGAACGAATCTGACAGTGACACAGAGCAACGACAGGGCTGCGATATTGAAGAAAGTAAACTTTTGTAATTCCGTAATATTTTGGATTAGCATGGTGGGAACTCTTCAGTACATGACCTCTCCTCTCCTGGAGACCCTAGTTCGAAGGTTTCTCATGAAGGAAGACTGCGAGTTGCTACTGCCAATCACTTGTAGCTATCCGTTAAGTCCATCTAATGATTGGGCGGTGTATATTTTAGTTTACGCATTTTTATTTTATAGTGAATGCCTGTGCGTGTTTGTCTACGTCGGTGCTGAACTGATTATGATAACCCTCTGCGCTAACCTCGGCATGCAGTTTACTTTGTTACGTGAAGATTTGCTTCAACTAAATCCGTTCGTCATGAGACATCAACAGTTGATTGAATTATCACGATTGTTGGATAGTGTGTTCAATCGAATTATATTCGTAAATCTGCTGTTTGTGGGTATAACAACGTGCTTCTTCAGATACGCCGGACAGTTCTCCCGAGGCCCCACGTACATGTTGAACAATTACGTAGCCGTGTTCTCATCTTTGCAGTTCGTTTTTTATCTTTGTTATTTCGGCGAATTGCTGACTGGAGCGAGTGCAAGAATCGGAGATACGGCTTACCAAAATCTGTGGTTTGAGGGCGGAACTCATTACCAGAAAACCATGTTGTTGATAATCAGAAGGTCGCAGCGCGCGTGCTGTCTTACGTCGTTGAAATATGCACCAGTCACTCTCAATATGTTCACGAAGGTGATCAGCACAACTTGGTCCTATTTCTCATTGATGAACACCGTTTATGGCGAACAAGAGTAG

>BmOR75

MEALKDYPVDFARSFKASLDFLSWSNIKFFAEDQTFIQRYWRLIITTPCVVLMYVTAILHICKLLYEEEIFVIAYLMPPVLVATQAILKAFVLVPNTSQVSHIMRELGSLWTRTNLTVTQSNDRAAILKKVNFCNSVIFWISMVGTLQYMTSPLLETLVRRFLMKEDCELLLPITCSYPLSPSNDWAVYILVYAFLFYSECLCVFVYVGAELIMITLCANLGMQFTLLREDLLQLNPFVMRHQQLIELSRLLDSVFNRIIFVNLLFVGITTCFFRYAGQFSRGPTYMLNNYVAVFSSLQFVFYLCYFGELLTGASARIGDTAYQNLWFEGGTHYQKTMLLIIRRSQRACCLTSLKYAPVTLNMFTKVISTTWSYFSLMNTVYGEQE

>BmOR76

ATGTACTACTACGCGATTCGTTCAAGGGTGACATTATCCAAAACGACTTCAATACTGGTTCCTATGACGTCACCGTTTTACGAAATCGGATTCTTGCTTCACGCAATATTCATGTTCGAAATAGCGTTTTTGCTGCTGGTGGTTGATATGTGGTTCGTTTTCTTTATGTTCTTTTACTGCATAGCTTGCGATAGGCTCGTGGAAATCTTGGAGGTCAAGAGAGATGTAGGAGTTTATGAGATTGAACTGAATAGAGCATTGAAAAAGTTTTACATTGCTCATGATAATCAGATGAAATATTTGAACATTATAAGTGCGATTTACAAATGGTCGACATTGATACCTTTATGTACAGTATTAGCGAGCATATGCATAATCATGCTTCAAATGACAGAGAAAATCAACTGGATCTTCGCTACTAATACGGTACCGACTCTAGCCGAGATCTTCGCTTACAATTGGTTTGGGGAAATGGTCAAGACAAAGGCTGAGAACGTCACATTGGCATTATTGGAATTCCACTGGACCGGTTTGCGTTATAAAGACAAGAAGAACTACCAAATTATAATATGCTACACCAACAAGGCTTTTGGAATTAGGACCGCATTCGGAAACGATCTGTCTATGTCCACTATGAGCGCTGTATTCAAAGCCAGCTATCAAGCGTTCGCGGTCATGAAATCGATGCAAAATTAA

>BmOR76

MYYYAIRSRVTLSKTTSILVPMTSPFYEIGFLLHAIFMFEIAFLLLVVDMWFVFFMFFYCIACDRLVEILEVKRDVGVYEIELNRALKKFYIAHDNQMKYLNIISAIYKWSTLIPLCTVLASICIIMLQMTEKINWIFATNTVPTLAEIFAYNWFGEMVKTKAENVTLALLEFHWTGLRYKDKKNYQIIICYTNKAFGIRTAFGNDLSMSTMSAVFKASYQAFAVMKSMQN

>BmOR77

atgacagtagcgtctatattgatagtagacaaaactgtctgtgttgatgtagacgtgtggacagttcaacgtgccctgtccggtctggtagcggcggtatacaatagaaatgactacgataaaacgttcgtaactccgaaggcgactctccactggagcggcatcaagatgacgcatacaattacagaattacaaggcaaatactggagcgtctactactggttcgaattcgtgaatattttcgctgcaatgtgtctcgagttcttaggcatgctagacacggcgagaggcgggacctttgacgacgcagtcaaaatatttcgtatgatgccctgcaatggatacactgtactttccctgataaaatccttcaatatggtacgataccgtccagtttacgagaatctaatcgacgagatcggtggaatgtggcccgacggcgcggtgggagaagaagaacacaagatcattagttcggcactcaagcaaattaacttcgttgttaaaggttattattactgcaatcactatttgctcgtgagcttcctgtgccctccattttttcaacggatcaaaggtctcttggggcgggagtgggagatgatgttgcactttttctactggctacctttcaaccctaaccagccggtatactttgagatcttgttgacaatacagacttggcatgcgattattgttatttggctgaacatgtccggagatctcctgttctgcttattcctgagtcacatcacgacccagctagacttgctctccgtaaggatcaagaagctggtgctcgtctccgtagatcggcagctgcctgatcattttcctctgggaatgctcagtaaggacactgagagactgagcgccgagcaagagatcaaggaacaacaacaagaactggctgagatcgtcaagcggcaccacgctctgatcaggttgtcaaaagacgtggaggatatgtatagcttctcattgctggtcaacttcttgaacagttccattttcatctgcttctgcggattttgcagtgtcgtggtggaaaaatggaacgaaacggcatacaagtctttcttggtgacaacactgtcacaaatctggttcctgtgttggtacgggcagaaacttctagactctagtgaaggagtatccgatgccctttacaaatgtggatggtacaacgcatcgaagaaagtcaaaaccagcatattgataatgctgcacagatcccagaagggcgttagtgtgacaacgttcggtttctctgtaatttcgctggctagttacagtaaaatcatgaagacagcgtggtcttatttcactttacttctcaatatctacaataagtga

>BmOR77

MTVASILIVDKTVCVDVDVWTVQRALSGLVAAVYNRNDYDKTFVTPKATLHWSGIKMTHTITELQGKYWSVYYWFEFVNIFAAMCLEFLGMLDTARGGTFDDAVKIFRMMPCNGYTVLSLIKSFNMVRYRPVYENLIDEIGGMWPDGAVGEEEHKIISSALKQINFVVKGYYYCNHYLLVSFLCPPFFQRIKGLLGREWEMMLHFFYWLPFNPNQPVYFEILLTIQTWHAIIVIWLNMSGDLLFCLFLSHITTQLDLLSVRIKKLVLVSVDRQLPDHFPLGMLSKDTERLSAEQEIKEQQQELAEIVKRHHALIRLSKDVEDMYSFSLLVNFLNSSIFICFCGFCSVVVEKWNETAYKSFLVTTLSQIWFLCWYGQKLLDSSEGVSDALYKCGWYNASKKVKTSILIMLHRSQKGVSVTTFGFSVISLASYSKIMKTAWSYFTLLLNIYNK

>BmOR78

ATGTCAGTCCGAGTACAGGAGCATTGCACGAAGGAAATCCCTGTAAAGAGAGGAGTGAGACAGGGAGATGTGATATCTCCGAAACTGTTTATCACTGCTCTGGAGGATTCCTTCAAGCTTCTGGAATGGCAAGGACTTGGCATCAATATTAACGGCGAATACATCACTCATCTTCGGTTTGCCGATGACATCGTGGTCATGGCAGAGTCGCTGGAAGATTTAGGGCACGAATTCTTAATTGAAATGGACTATATTAGCAAAATTGGTTCAAAGCTATTTGTTTATCCTTTCGTGGGACGGTCAAAGTTTACTATGTGTTGTTATTATGTGACTTTCTTTTTTCTTGTACTTACGTCGGTCCAATTATTCGTTACTCTATGTTTAACCCGATTTGAGAGCAGCTTCGAGGTGATAAACATAGCTCCCAACTTGGGCGTCTGCCTTGTGATCATAATAAAATATTCAAAAATTCATACGAAAAGGATTTCATATCAGAAATTTTACAATCATTTTCGTTACGAGCTATGGGATGTTGTTTTGGATTCGGTTGACCACAGGAATGTTCTAGAAACATATATGCTTTACGAGAACAGCGTGTTGCAAAAGAACCTGTATTTGTATCCATTCGATGGATGGTACCCCTTTAACAAGATCAAGTGGTACTATATCGCTTACGTTTGGGAGAGCGTAATGACTACCGTCGTTATAATTAACTTCGTGTGCACGAATACAATCCATATTTCTTACACGAGACTCATTTGCATGGAACTAAAAGTTCTCGGCATCAGCATAGAGAATCTATTAAAATCCAAGGTTCGAGATTCGATAACACAAAGTAAAATTGAAGACTTCCACGAAAATATAAAAGTGAATTTTAAAACTATTTTAAAACGGCATCAGCTCTTGGGAAATGTAGTATCAGAGTTAAACATCATAATGGGAGATGGAATGCTTTTGACTTACATCTCTGGATCTGTTTTCATTTGTCTCACAGCATTTACTGCAACCGTCGTCAATGACTTTTACATGACTCTGCGTTATTTTTCATTTTTCTGCTCGTTGCTGGTCGAAACCTTCATCCAATGTATTATGGGACAGCTTCTGATAGATCACAGCGAAGATTTTGAAAATTCTATCTATTTTACCGATTGGCCTATTGCTGATTTAAGCACAAAGAAAATGCTTTTAATAATGTTAATCAGGGCCCAGAAATCATACGTTTTCACCGCTAATGGATATTTTATTATGAACTTTGACACTTTTGGTGGGATTTGCAGTCTCTCCTACCAACTGTTTAATCTTCTACGCACAACTTATAACAAAGAACTTTAA

>BmOR78

MSVRVQEHCTKEIPVKRGVRQGDVISPKLFITALEDSFKLLEWQGLGININGEYITHLRFADDIVVMAESLEDLGHEFLIEMDYISKIGSKLFVYPFVGRSKFTMCCYYVTFFFLVLTSVQLFVTLCLTRFESSFEVINIAPNLGVCLVIIIKYSKIHTKRISYQKFYNHFRYELWDVVLDSVDHRNVLETYMLYENSVLQKNLYLYPFDGWYPFNKIKWYYIAYVWESVMTTVVIINFVCTNTIHISYTRLICMELKVLGISIENLLKSKVRDSITQSKIEDFHENIKVNFKTILKRHQLLGNVVSELNIIMGDGMLLTYISGSVFICLTAFTATVVNDFYMTLRYFSFFCSLLVETFIQCIMGQLLIDHSEDFENSIYFTDWPIADLSTKKMLLIMLIRAQKSYVFTANGYFIMNFDTFGGICSLSYQLFNLLRTTYNKEL

>BmOR79

ATGGCGGACACGTTCTCTGCTTTTCACCGAGTTCTATCCTTTGCAGGAATATCAATCTTCAAAGAAACCAATTGGAACTCAAAATTCTGGTTCACCGTCCAAATATTCAACTTTATCATTGGGCTGCTTTGTTTCCTCTTCACCTCTGGTTTCGTAATTAGCAGTTCCTCTGACCTTCTTCTTTTCATTCAGGGCGCTTGCATTTGGACCACCGGTGTCATTATGACAATAACTCTTGGAATATGCTTAGTTTTTCGCATCCAATTCCGAGATTTTCTAACCGAGATGGCTTTTAGAGATGCAGCATTGGATATACCTATCGTTGATTACATTTTCAATGTGAATAATGGCAAGAAGATGATGCAGCTTAAGAAATTGGTTGTAGAGTCGCAGAAAGATTTGTTAAATTATACTAGTTTACTGTTGAAGATATATGTAACGGGAGTGTGGCTTTGTGCCACACTGTATCTCTGCAGTCCAATTTACAGTATGCTTGTTAGTGAAGACAAATCTTTGAGGCTGCTAGCATTCGACATGTGGTTTCCCTGGAGTTTGGCGGATATAAAAGTGTACACCTTGTCATTTATATTCCACGCATATGCTGGCTACTTATGTTGCGTAGCTTACCCCGGTCTTCAATCAACGATAATACTACTGGTCGGTCAAATTATTCGTCAATTGAGGATATTAAATTTCATTCTGCAACACTTAAACGAGCTGGTGCTAGAAGTTAACCAACGGAAAGATGAAAAATGGCAAAAATCTTGTACTACGGTCCTGTCTCAGTGTGTTGAGCATTACGTAAAAATTAAAAGATTCAGCAATCGCCTAAATGTAATATGCCGCCCTTTCTATTTGGCGTTGATTCTTGTGGCCATTATCCTTGTTTGCATGTGTTCGGTGAAGATTGCTATTTCTGATAAGCTGAGTCCGGATACAATTAAATATTACGTGCACGAAATCTGTTTTATCTTCGTCGTGTTAATGTTTTGTTTATTGGGACAGCAAGTCGATGACGAATGTGCATCCCTAGAGCTTGCCGTCACAGAGAAATGGTATATCTTCAATCGCGCTCACAAGCAGAATGTCCGAATATTCAAAATGGCGCTCAGTCAAAGAATGCCTATTTACATCTTCGGTACTATCACCCTATCACTGCCAACATTTACTTGGTTCATCAAAACTGGGATGTCTTTCTTCACTTTGGTAATGTCCGTTTTAGAAGAACAATAA

>BmOR79

MADTFSAFHRVLSFAGISIFKETNWNSKFWFTVQIFNFIIGLLCFLFTSGFVISSSSDLLLFIQGACIWTTGVIMTITLGICLVFRIQFRDFLTEMAFRDAALDIPIVDYIFNVNNGKKMMQLKKLVVESQKDLLNYTSLLLKIYVTGVWLCATLYLCSPIYSMLVSEDKSLRLLAFDMWFPWSLADIKVYTLSFIFHAYAGYLCCVAYPGLQSTIILLVGQIIRQLRILNFILQHLNELVLEVNQRKDEKWQKSCTTVLSQCVEHYVKIKRFSNRLNVICRPFYLALILVAIILVCMCSVKIAISDKLSPDTIKYYVHEICFIFVVLMFCLLGQQVDDECASLELAVTEKWYIFNRAHKQNVRIFKMALSQRMPIYIFGTITLSLPTFTWFIKTGMSFFTLVMSVLEEQ

>BmOR80

ATGTTTGAAAAGATAAAAAGTATTTATAATAAATCAAATTATGATATTTCTTCAGAATTTGTGAATCCTTTGGACTACCATAAAACTTTTTATTTTATATTGAAACAATTTAGAGTCGTAGATGTGGATTCCTCTAAAAATAAGTACTGCAATCAAAATATAATTCTTTTCACAATTGGAGCTATAGCAAACACATTGATGTTAATATCTTTATGCCATGGAATACACAAAATGGAAATACCACACATAACTGAAGCTGGAACCTATTGTATTGTGTTGAGTTATAAGTTATTGATCTTGTCATGTACTAAAATAAATGTTGTGCACTACGAAAATTTATTGAAGTCTATGAAAGAAGACTTCAAATATATATTCACAAATGGCAAGAAATATAGAGAACGATTCTTCGGTCTACAACTGGTAACATGGAAAATTTCCCTATTTTCGGTAATATTTACTTTTAGCATTCCTGTTGGCATGATTATATCTGCTTTCGGGTCACTGTTATACTACTTATTAACAAACGAATCAAGAAACGGCAACAAGAGACCTCTGTTATTCCCATTTTATATTCACGGCGTAGATTTTGGTGATACCCCAATTTATGAAATTGCTTTTACGTTTTCAAACATCTGTACTTTGGCATACGCTTATAATTATATTTTTATGATACAAACACAAATAGTGTGGGTGAGGCAAATAACATCAAAGGCGGATATTATAATATGGAACCTTCAAGATCTTTTAAGAGATATTTATCCTCCTATAAATGAAACACAGAGGGCTTATTTTTTAAATCTGATTAAACATCGAATGAGGGATATAGTAAGACAGCATCAATCGATGTACAGTCTAATGGAAGATTACTCACAAGTTTACAAAAAGTTGCTGCTTTTTGAGCAAAAATGCTGCGGTCCAGTAGTTTGTCTTACTGCATATTGTGCTACTGAGAAACTGGCTGAAGGGGAACTAAACGTAGTTCTCATTTTACTCTGTGTCGGCACGATTGTAATGCATTACATCCCTAGTCATTTGTGCACGTTCTTAACAATAAAGGTGCGTTCAATTTGCGACGCTTGTTGGGACACTCCATTCTGGAATGCGGATAAGGCTATCAGACCGTACATAGTTCTGATCATGCAAAGATCCCTAAGACCCCTACCTTTGAGAGCAGCAGGTTTCGAAGATATTTGTATTCAAACTTTTTCTAGGAAAATGACGTCCGCTTATTCGTACTTTAATATGCTGAGGCAAGCTAATCGCAAATAA

>BmOR80

MFEKIKSIYNKSNYDISSEFVNPLDYHKTFYFILKQFRVVDVDSSKNKYCNQNIILFTIGAIANTLMLISLCHGIHKMEIPHITEAGTYCIVLSYKLLILSCTKINVVHYENLLKSMKEDFKYIFTNGKKYRERFFGLQLVTWKISLFSVIFTFSIPVGMIISAFGSLLYYLLTNESRNGNKRPLLFPFYIHGVDFGDTPIYEIAFTFSNICTLAYAYNYIFMIQTQIVWVRQITSKADIIIWNLQDLLRDIYPPINETQRAYFLNLIKHRMRDIVRQHQSMYSLMEDYSQVYKKLLLFEQKCCGPVVCLTAYCATEKLAEGELNVVLILLCVGTIVMHYIPSHLCTFLTIKVRSICDACWDTPFWNADKAIRPYIVLIMQRSLRPLPLRAAGFEDICIQTFSRKMTSAYSYFNMLRQANRK

>BmIR1.1

ATGTGGCGAACTGTAATACTTGCAGCGTGTCTCACTCCAGACCCTGGCGCGTCTCTCGGAGCCGGCAGCGCCGCCGTGGATTATTTTGTGCAAAAGGGAGCTCCCTACCTCTGCTACTTGACATGTGAAAACAAAAAGTTAGTACACGAGTTCATGGACAGAGGCATCAGAGTTTCGTTGCAGCTAATTGATAAAAGTTGCGATCTCGAAAGGAACTTGCTGCAGTGGAATGTACCTGTTGGAGTCCTGTTGGATGCTGGGTGCGAGAATACCGAAGATATATTAAATACGGCATCTCGAGGTATTCTGTTTGACGCCATGCATATGTGGCTTATTAAGGAGATTGCAGACCACGAGGGCGGGAATATTCTGAACGTGTTACAACGAATGAACCTCAGCGTAGACGCTGATGTCGTCGTTGCTTTTGATAAAGGCGACCGCGTAGAACTAATCGATGTTTTCAACTATGGGAGGATACAGGGAAATGATTTGGAGAAGAAATATATAGGGGAGTGGAGTCCAGAGGGCGGCCTGAACGTTTCGCTGAACAGATTCAAGTATTATGATCGGTGGGATTTCCACAATTTAACTCTGCGAGCCGTGACTGTGATTTTAGGCGACCCAAAAGATTTCATGCCTGAAATGCTATCGGACGTGGGATATAAAGCCGGAGTGCCGCTCTTGACAAAAATACCGACACAGCTCCTGTATATACTGAAGGATATACATAATTTTAGTTTCAAGTACACAGTGGCGGGGCGCTGGATCGGAGCGCCGGAGAGGAACTCCACTTTGGCGGTCACCAACTCCCTATTCTGGGGAGAACAAGACATCTCCTGCACTAGCGCGAGGATGTTCAAGCATTGGCTGGAGTGGGTCGATGTGTTCTCCCCGCCTGCTACTAGTTTTGAGACAAAATTCTACTACCTAATATTAGACCAAGGCATAGGTAGCTACGAGAATAGGTTCCTGACGCCACTGTCAAGCGGCGTGTGGTTGTGGACGGCAGCGGCGGGAGTCGCTTGTACAGTACTACTGGGTGTAGCTGCGGTGCTAGAGGCAAGAAGCGAGCCCGGATTGTATGCATTTTTTAGCGTTTTTGCTGTTATTTGTCAACAGGCATATGAAGATGGAGTCAATTTATTTGAAGAAATGTCCTCGAGTCAAGGTCGTCGTCTTGTCCTCCTCGTCGTAGGCATAACTAGTATGTTACTATACAACTACTACACGAGCAGCGTGGTCTCGTGGTTGCTGAACGCGGCCGCGCCCACCCTCGCTGACATGGACGCGCTCATCAACAGCGACCTTGAACTGGTCTTTGAAGATATTGGATATACCAGGGGGTGGCTAGATAACCCAGGCTTCTTCTACTACAGCGGCTACAAGAACCCTAAGGAGGACGAGCTCCGGCTGAAGAAGGTGACGCGAGCACGGCGCACAACCGCGTTATTGCAGCCCGCCGCCGCTATCAGCCTTATACGGACTGGGACCTACGCGTACCACACGGAGCCCTACACCGCCTACCAGGTGATCTCGCGCACGTTCTCGGAGCGCGAGCTGTGCGAGCTCGGCGCCTTGCGGATGCTGGGACCCGATAACGTGTACATATTCGGTCAGAAACGGAGTCCGTATAAACAATTCTTTGTTTGGAGTCTCATGCGTCTCCTCGAGCGTGGTCACACATCTTTAGCGAGCGCACGCGTGTCTGGTCCACGTCCTACATGCTCGGGGAGAGCGCCGCGCGCACTCGCTTTAGGGCAGGCGGCGCCGGCGTTCGCGGCGTTAGGTTACGCTGCGTTGCTGTCTGTTATTATAGCTGTAGTGGAGGTTTGGTTTCACAAAGCGCAAAACAAAAAACAAGCCCGCGGAGAGTTCGATAATCGCGCACATAACGCAGAGCGGAGTGATTTTTTTAGCGAAGCATAA

>BmIR1.1

MWRTVILAACLTPDPGASLGAGSAAVDYFVQKGAPYLCYLTCENKKLVHEFMDRGIRVSLQLIDKSCDLERNLLQWNVPVGVLLDAGCENTEDILNTASRGILFDAMHMWLIKEIADHEGGNILNVLQRMNLSVDADVVVAFDKGDRVELIDVFNYGRIQGNDLEKKYIGEWSPEGGLNVSLNRFKYYDRWDFHNLTLRAVTVILGDPKDFMPEMLSDVGYKAGVPLLTKIPTQLLYILKDIHNFSFKYTVAGRWIGAPERNSTLAVTNSLFWGEQDISCTSARMFKHWLEWVDVFSPPATSFETKFYYLILDQGIGSYENRFLTPLSSGVWLWTAAAGVACTVLLGVAAVLEARSEPGLYAFFSVFAVICQQAYEDGVNLFEEMSSSQGRRLVLLVVGITSMLLYNYYTSSVVSWLLNAAAPTLADMDALINSDLELVFEDIGYTRGWLDNPGFFYYSGYKNPKEDELRLKKVTRARRTTALLQPAAAISLIRTGTYAYHTEPYTAYQVISRTFSERELCELGALRMLGPDNVYIFGQKRSPYKQFFVWSLMRLLERGHTSLASARVSGPRPTCSGRAPRALALGQAAPAFAALGYAALLSVIIAVVEVWFHKAQNKKQARGEFDNRAHNAERSDFFSEA

>BmIR1.2

ATGTGGTATTTAGCAGTAGTGATATCAATATTTCCTTGCCTGATTTCAGGCCTAGATCAGAACTCTTTGAATTTTGGTATTGAATATTTAAGGTACCGAGATTTGAAGTTTGTCTGCTTGCTGACATGTGAAAAAGATATTTCTTGGGCTCTTCAATTCTCAAAATCGTCATCGCGTTTCATGATGGCAGTGTCAGGGGCTTTTATAGACGACTCTATGTCTGACTTCGAAAGAGTCGAAAATTGTTTGCAGCGTAAGCTATACCCTGTTGGAGTTCTCATAGATTCTGGATGTGGAAAAACAGAAGAAGTGATGTATTTTGCATCTCAAAACATGTGGTTAGATGGCAATCACAAATGGGTCCTTATCAACGATGATGGCTCAGAAACTGAGAGGTACGGGGATGTTGATAACAATACGGTCATGTTTGATAACGATAAAAATTCGAGCATCATGGATACTGGTATAATGGAAGTACTTAGTAACCTGAATATAAGCGTCGATGCTGATATTGTGGTGGCTGAGAGAGATAATTCAAACTACATTCTCTACGATATCTTTAACTATGGCAAGATTCAAGGAGGCAATTTGAACGTACATGAAGTTGGATCGTGGGGACCTCATAATGGCTTTAATTTGGATATCAATTTGAACGGGTACAAGTATTACCGACGATGGGATTTCCAGGATATAACAATGAGGATGATTTTGGTCGCTCAACCAGCTCCAAGGCATTTTGATCTGGAATCGTTAACAAAACCAACACCAGTTCCTGGAGTAGCCGTGATCACACAAATAAGTACTGCTGTCCTGTACGATGTCGCTAAAATGCATAATTTTCGCTTCACTCCCACCATTACAGACAGATGGATTGGGGAATACGAGAAAAACAGTTCTAAGGTGGTCACCAACGCCTTATACTTTCGAGAACAAGACATATCACCTACAATTCGACGTCTCAAAGCCGTTCAAGAACACAACGATGTTTTGAACTCGCCATTGACTGCAATTGAAACTCGCTACTACTACCGCATCCCAACTAAGGGACCTGGAAAGTACGAGAATCAATTCCTTAGACCTCTAACACCAACAGCATGGTGGGCTGTGATAGCAGTATCAACTCTATGCGCTTTTCTACTTCTCCTGTCAGCAATGTCAGAACAACGTCCATCGTCGCTGCAGTATGCAGTGTTCTCTGTCGTTGCTTCTATTTGCCAGCAATTTTTCGAAGATATTGACGACGGTGGAACAAAGAGAATTTCAACAGCTCGGAAGGTCACAATCCTGGTGACAGGTCTGTCCTGTGTACTGCTGTATAACTACTACACCAGTTCCGTGGTCAGCTGGCTTCTGAACGGTCCACCACCTTCCATCAACTCCTTGAAGGAACTGCTCGACAGTCCGCTGGAGCTGATATTTGAAGATATTGGGTATACGAGGTCTTGGCTACAGAATCCTTTATATTACTACAACAAAAGAAACGCCTTAATGGAAGATGAGCTCCGTAAGAAGAAGGTATTGAATAAGAAGAAGAATGCTCCATTGCTGGTAAACCTGGTGGAAGGGATCGAGATGGTTCAGAGAGGAGGCTTTGCTTACCACACGGAAGTGAACAGCGCGAATGCACTGATCTCCAAAACATTTACACAAGACGAGCTATGCGAGCTGGGTTCCTTACAATCCATGGAGAAGACATTGTTATATCCAGTTCTTCAAAAATATAGCCCGTTTAGGGAGTTTATGAATTGGAGTATCAAACGTCTAACAGAGCAGGGTATAGTCTCTTGCATCCAGCTCCGAAGGTCATCATTCGAGGTGAAGTGCGAGGGAAGTTCTCCTCGGGCCCTCGCTCTGGGAGGAGCAGCTCCTGCCTTCATACTCCTGGCTGGAGGCTACTTCTTTGCTACAGTTATTATGCTGATTGAACGATACGTTTACAAAATGAAACATAGCCACGTTATCTTGAAATAG

>BmIR1.2

MWYLAVVISIFPCLISGLDQNSLNFGIEYLRYRDLKFVCLLTCEKDISWALQFSKSSSRFMMAVSGAFIDDSMSDFERVENCLQRKLYPVGVLIDSGCGKTEEVMYFASQNMWLDGNHKWVLINDDGSETERYGDVDNNTVMFDNDKNSSIMDTGIMEVLSNLNISVDADIVVAERDNSNYILYDIFNYGKIQGGNLNVHEVGSWGPHNGFNLDINLNGYKYYRRWDFQDITMRMILVAQPAPRHFDLESLTKPTPVPGVAVITQISTAVLYDVAKMHNFRFTPTITDRWIGEYEKNSSKVVTNALYFREQDISPTIRRLKAVQEHNDVLNSPLTAIETRYYYRIPTKGPGKYENQFLRPLTPTAWWAVIAVSTLCAFLLLLSAMSEQRPSSLQYAVFSVVASICQQFFEDIDDGGTKRISTARKVTILVTGLSCVLLYNYYTSSVVSWLLNGPPPSINSLKELLDSPLELIFEDIGYTRSWLQNPLYYYNKRNALMEDELRKKKVLNKKKNAPLLVNLVEGIEMVQRGGFAYHTEVNSANALISKTFTQDELCELGSLQSMEKTLLYPVLQKYSPFREFMNWSIKRLTEQGIVSCIQLRRSSFEVKCEGSSPRALALGGAAPAFILLAGGYFFATVIMLIERYVYKMKHSHVILK

>BmIR31a

ATGTCTATTGCGAATATATTACTGCTCATATTAGCGGGTGTACCTACCTATTGGACAACAATGCTCGTTCAAGCTATAGCAGATTTTTTCAAATATAAAATAATAAACACTGTAATTGTCTTGTCTTGTTGGTCTTCTAATGATCGTGTTAAATTTATGCGTCAATTATCTGATCATGGATTAATCGCCACAATTTCTTGCGACCCAACTATACTCGATTATGTTCAGAACCATCATTTTCAAGGAATACTATATGTGAAGCAAGTAAATGATAGTTTGTTGGAAAAGGTGAATCCGGTCTATTTTTCCAACTGGTATAAATGGCTCGTAATCAGTGACGAAGCACCCAGCAGTCTACACGCGACTCGTTATGATGCTGATGTGGTTCTTATTGAGTCATCAAAGAGAATTATGGCACGTGCAACAGGAAATAATGAAATAATGTCCACAGCGGCAATAAAGCAAACTATTTACTTTAATGATGTCTACGTGCATCCTCGAAATGGAGCTTCTTTGAACCCGTGGGCAGTTTGGACTGGAACTCTTGAAGTCACACATGAAAGGGAGAGAATTTTACGACGACTTGATTTGAAAAAGTATCCGTTGAGAATCGCAACTCCTGTGGGTCATTACTCTGAAGACACTTATAATGGTACTTTTGAGGAATATCTAGCAGATAACACCATGCCCGAGCGTGATTCAGCTACTCGCTGTGGGCATGCTGCTTCTTCACTAATTCTAGAATCTTTAAAAGCTACTGAGGTTTTAACACCGACGCTGTTGTGGGCCACTGAATTGAATAACAGTAGTATGATGTTGAGGGTGGCTTCTGGTACAGCTGAAATAAGTGGCTCCATCTTAAGAGTTTTGCCTGAGCGTATTAAGCGCCTTGACTACGTTATGCCGATATGGCCTTTCAGCGTCGGCTTTACATATTTAGCCGAAAGGGCGAGCAGTAGCAACATGTTCGTGGAGCCATTTTCACCCGGTGTTTGGTGGACATGTCTCGCTATTGCTGTTTTATTATCATTTGCTCAAAGACTCACAGCTAGAGAGCCGATGGAGAAAGAAGGCGCTTACATCGCTGTATTGGCGACTTGGTTGCAACAAGATGCTAGCGCAGTACCAGAAGGGGCATCAGGCCGGTGGACGTTCATCGTTCTCTCTGTATGTTCGATGCTAGTACACGCTTACTACACCTCGGCCATCGTCTCAGCTCTCATGAGTACCGGCAGGAGTGGACCAGATTCGCTGAAAGCCCTTGGCGATTCCAAATACGCTATAGCTTCGGAAGACTACGACTACATGCGTTACACTATGTTTGGTATGGAGACCAACTGGGACGATTTGGAATACCTCAAGAAAAAGAAAATGCATTCGAATTTCTATCAAAATATAGAACGTGGAGTGGAACTCATACGAGAAGGGAATACCGCTTTTCACACAGAGTACAATCACATTTACCCGCATCTGAGGACATTCAATGATGAACACTTATGCAAACTTGCTTACGTTGATACTATTCCAGAGATAATGACATGGATAACAACAACTAAACGCTGCCAATGGACAGACGTGCTGCGTACTGCTGGCGGATGGCTAAATGAAGTAGGATTAGTGAAACGTTTGGTATCTCGCTGGCGAATACGTCCACCGCCATGCCGAGCTTCATTACTTGCAGAAAGAGTTAAATTCGGAGACGTAGCTCCAGTATTATGTCTAACTGCTATAGGTGCCATAGCATCATTGATACTATTAGGACTAGAAATAATATTTGCTAAATGGACAGGAAGTAAATATAGAAATTCTCCCGTCAGTGATGTCGCTGATGTTGCAAGTGGAGACGAAAATATTAATAAATAA

>BmIR31a

MSIANILLLILAGVPTYWTTMLVQAIADFFKYKIINTVIVLSCWSSNDRVKFMRQLSDHGLIATISCDPTILDYVQNHHFQGILYVKQVNDSLLEKVNPVYFSNWYKWLVISDEAPSSLHATRYDADVVLIESSKRIMARATGNNEIMSTAAIKQTIYFNDVYVHPRNGASLNPWAVWTGTLEVTHERERILRRLDLKKYPLRIATPVGHYSEDTYNGTFEEYLADNTMPERDSATRCGHAASSLILESLKATEVLTPTLLWATELNNSSMMLRVASGTAEISGSILRVLPERIKRLDYVMPIWPFSVGFTYLAERASSSNMFVEPFSPGVWWTCLAIAVLLSFAQRLTAREPMEKEGAYIAVLATWLQQDASAVPEGASGRWTFIVLSVCSMLVHAYYTSAIVSALMSTGRSGPDSLKALGDSKYAIASEDYDYMRYTMFGMETNWDDLEYLKKKKMHSNFYQNIERGVELIREGNTAFHTEYNHIYPHLRTFNDEHLCKLAYVDTIPEIMTWITTTKRCQWTDVLRTAGGWLNEVGLVKRLVSRWRIRPPPCRASLLAERVKFGDVAPVLCLTAIGAIASLILLGLEIIFAKWTGSKYRNSPVSDVADVASGDENINK

>BmIR40b

ATGTTCAAAGAAATAATCCGCATTTGGCCCAGCAGATGCGTCGCTATGCCCTCCAGGATATCGAAGAGGGTATTCAACGGCTTAGGTCGAAACGGAACACTAGATCTCTTGATATCAGACACTCCTGTTTTGGATTACTATAGAGCAACCGATCATGGATGTAAGCTTCAACGTGTGGGCGGTCGTACTCTGGCAGAAGATACCTATGCTATTGGCATGACAAAAGGATTTCCATTGAAGGACAGTATATCTTCAGTTATAGCTAAGTATTCGTCGAATGGATATATGGACATATTAACTGAAAAATGGTACGGCGGTCTACCTTGTTTTAAACTCAGCCCAGATTATGGCATTCAACCGAAACCTTTAGGTGTGGCGGCTGTGGCTGGTGTTTTTATTCTTCTTGGAGTAGGAATGATCGTTGGAATTATCATTTTGATTTTCGAACATCTGTTTTATAAATACACTTTACCTATATTAAGGCATCAACCGAAAGGAACAATCTGGAGGAGCAGGAACATTATGTTTTTCAGTCAGAAACTATACCGATTTATCAATTGTGTTGAATTGGTATCGCCGCACCATGCCGCCCGAGAATTAGTCAATACAATACGCCAAGGTCATTTCACTTCTCTGTTCCAAAAGAGCGTTAAAAGGAAGGAACATGAACAGCGCAGGCGTCGTAAGAGTAAAGCGCAATTGTACGAAATGATTCAAGCGATTAGAAGAGTACAACAACGGGATCACTCTTTGGGGTCTATTAAAGAACAAGAGCCAGTTGATACATCTGAGACAACAGAAGAATTAACTGAATCAAAGTTTTTATCCCCGTCACCCGACACATCGCACCGCTCACCAAGACAAGGTCGTTCACCTCGACAGCTTCGTTCGCCTAAAGGTCGTCGAAAAAGATGCAGTCTCGCAGGATTAAACGTTAGGAGATTTAGTACCGATTCAGTGTTGGGTTCAGATTCTGTTTCCAATATTTATGAGAGAACTTGCCACAACATAGGCAGACGACTTAGTCGAGATGTCAGCTGCTTAACAAATTCCCCTCCAGATTTAAACACCCGATTGCGAACACCGTCTCCGATGATAAGACGGACCGAAGCTTCTTCAACCCGTTCTTACCAAGACGTATCCTTAAGATCCGAAAATTATGTTAGCACAGATGCTCCAACGTCTCGAGCGAGTATAGATATTTTAATTTCTGATGAACATGATATACCTCCTGCGCCACCATATCCTAGAGTTTCTCCGGCGGGTGCTCGATCCGAACTTTCCTTACTATCTGAAGAAGAGTTGATTAGACTTTGGCGAAGTTCAGAACGCGAAGTCAGAGAGGCTTTATTAGCAGCACTACAAGAAAGACGGGCGAACTTAGATCCCAAAGAAGATCCGGGATGA

>BmIR40b

MFKEIIRIWPSRCVAMPSRISKRVFNGLGRNGTLDLLISDTPVLDYYRATDHGCKLQRVGGRTLAEDTYAIGMTKGFPLKDSISSVIAKYSSNGYMDILTEKWYGGLPCFKLSPDYGIQPKPLGVAAVAGVFILLGVGMIVGIIILIFEHLFYKYTLPILRHQPKGTIWRSRNIMFFSQKLYRFINCVELVSPHHAARELVNTIRQGHFTSLFQKSVKRKEHEQRRRRKSKAQLYEMIQAIRRVQQRDHSLGSIKEQEPVDTSETTEELTESKFLSPSPDTSHRSPRQGRSPRQLRSPKGRRKRCSLAGLNVRRFSTDSVLGSDSVSNIYERTCHNIGRRLSRDVSCLTNSPPDLNTRLRTPSPMIRRTEASSTRSYQDVSLRSENYVSTDAPTSRASIDILISDEHDIPPAPPYPRVSPAGARSELSLLSEEELIRLWRSSEREVREALLAALQERRANLDPKEDPG

>BmIR68b

ATGAAGTTGGATGAACTGTTACGAATATTTCTTATAATTTTTCATATTTGTGATCATTTTGCGACACCACAAGGCTTAAATTCTAATATTTTTAAAAATAGTTCTCTACATGATACTTACCAAAATTCAGCAATCGACTGTATTGTGTCCACAAGCTACAAGCACATTCCGTGTGGTACTCTGTTTACATTAGTATATTCTTCTTANNNNATTAATGATGTTAATAAGCTTTTAAGTAACAAAAGCTGCTATTCATTTCTATCTCGATCGTTTGAAATCAGAAAATGGAGAACATGGACGGATGTGTACATATTTTTTACGAAAAACTATTATGAAATGTACACAAGTTTTACCACATTGTCAAAAGATGTTGTTTGGAATCCCAGAGCGCATTTTTTTATAATCATTAATGATCTACTTGATGCGGAGTATTCACAAGTTTTCAATATATTAAGGAATCTTAACATATTTAACGTTTTAATTATAACAAAATCAGAAAAGGGCAAATTCAGCGCGTATACCTATCACGCGTTTGAAGAAAGCAATTGTGGACGGTCACTTAATAAAATTGAAAATATTAATGACTGTAGAAATATATCTTCCATCAGAATGAATTACACACACTTAAACTATCAATTTCGAAATTGTGTAATATCTATAGCAGCAACTGAAGACATGCCTTACGTCATTTTTGAATCAAAAGAAGTTAATAAGCGTCCTAGAAAGATAGAAAAAGGAATAGAACAATATATACTAGACAATATAGCAGAACAAGAAAATCTTACGCTACAATATATTTATACAGATTCGAAACATGAATTTGGGGTTATTTTATCAAATTCAACAATAACAGGTTTACTAGGATACCTACATAATAACACAGCTAGCATTGTTATCGGAGGTTATATGTTAATAAGAAATCGAATCAGTTTATTTGAATATATTTGGGGTTATGATACAGCAAGTCTTTGGATCTTTACTCCAGCTTTGCGCGAAGAAAATTGGAAAAAAGTTTACCAAGAATTTGGTGTTTGCACGTGGCTATTAATAGGTTTCTCATATATATTTGCAACGGTTGTCATTACCACGTCACGTCGTCTGTTAATAGGACACGAAGATATAAGAAGAATAATCTTAAAAATGTGGGGTTACCTATACGGTCAGACAGACATTGAGTTGATTAAATTAAAAAAGATGAAAAAAATTGTAATATTTTGGATTTGGTTCACCTTTTTTATGACAAGTTTTTACAATTCAGCACTTTACAGTTTATTATCGAGAAACGTCGAAGTAAGATCAAAAATTAACACCAAGTCATTATCTACGCTGCCTTGGGATCCTTGCATAAGTAGTGGTATAAGAACTTTTTACAAAATCTCTTTTAACGAAACACTTCCTGGATATACAAAAGCAAACTGTATACTGACTGATGATGTACTAGACACTGTAGCTAACAATAATAAGTATTATACTTTGGAAATGGACTACAGCTACCAGTTGAGAGAGCACTACTATGTGGATGAAAATGGTAACCCAAAATTACAAGGTTTGCAGTTTGCAAGTGATATGATGCACGCAATGTACACCACTCGAGGATTCCCTCTACTTCATAAATTTCAAAGATATGCCACTATTCATTTAGAATCAGGGCTATTGCAACGACAACGTTCAGCAATCTCCCGCCATTATGCAACTCCTAACCATCGTCACAAAAAAACGTTTAATATATTTCATTTGTCAGATTTTAGAATTCATTATGCTATATTGGTTTTTGGATATACTATATCATTTGTATGTTTTATTATAGAAATAAATCGCAAATAA

>BmIR68b

MKLDELLRIFLIIFHICDHFATPQGLNSNIFKNSSLHDTYQNSAIDCIVSTSYKHIPCGTLFTLVYSSXXINDVNKLLSNKSCYSFLSRSFEIRKWRTWTDVYIFFTKNYYEMYTSFTTLSKDVVWNPRAHFFIIINDLLDAEYSQVFNILRNLNIFNVLIITKSEKGKFSAYTYHAFEESNCGRSLNKIENINDCRNISSIRMNYTHLNYQFRNCVISIAATEDMPYVIFESKEVNKRPRKIEKGIEQYILDNIAEQENLTLQYIYTDSKHEFGVILSNSTITGLLGYLHNNTASIVIGGYMLIRNRISLFEYIWGYDTASLWIFTPALREENWKKVYQEFGVCTWLLIGFSYIFATVVITTSRRLLIGHEDIRRIILKMWGYLYGQTDIELIKLKKMKKIVIFWIWFTFFMTSFYNSALYSLLSRNVEVRSKINTKSLSTLPWDPCISSGIRTFYKISFNETLPGYTKANCILTDDVLDTVANNNKYYTLEMDYSYQLREHYYVDENGNPKLQGLQFASDMMHAMYTTRGFPLLHKFQRYATIHLESGLLQRQRSAISRHYATPNHRHKKTFNIFHLSDFRIHYAILVFGYTISFVCFIIEINRK

>BmIR75p.2

ATGTATCTCTGGAGAATTCCGCTGTTGATTACCAGCGATTTAGTGCTGGCGCGAAGATATGGAGACCTCTTTGAACTTGTAGAACTACACAAACCAGCTCCAAGTGGTTCCATGATAACCACACCGCGCGGTTTCTTTAATGGTTCGCTAATAGACATGCGCCCTCAGAAAGAGCTCTATAGGAGACGCAGAAACCTAATGGGCCATCATCTGACCATGGCCAATATCATACAAGACAGTAACACCACGAAATACTATTTACCGAGGGATGATAGACTGCAGTTACACCACGACGCAGTTGCTAAAGTCTGCTGGGTTTTTGCGAAAGTGGCCTTCGAGCTACTAAATGCGACACCTCGGTACATATTCAGCTACAGATTTGGATATAAAGTGAACGGTCAGTGGTCCGGAATGGTACGGGACATAGGCGATAATAAGGCTGATTTGGAATACGATCTTCACTATTCACTGTCTCTCAAAGGAACCAGCAGCATGCATTTCAAAGAACGTCTCGAGATAATTACGTACACGGATCTGGTAGCACCGATGCGGATGCGCTTTGTGTTCAGACAGCCACCGTTGGCATACGTCGCCAATATTTTCGCTTTGCCGTTCTCAACTGGAGTATGGATGGCGGTAGCGATAGCTTCAATTGCGTCGACAGTGACGCTATACATTACCAGTCTGTGGGAGATTCGAATCGAAAAGAATCCAACTCAACTAGACGGCAGCATCGGAGATGCATTACTGCTGACAATGAGTGCTGTCACTCAACAGGCTGTCATCGAACCAAGAAGACACCAGGGTTTATTCGCTTTCCACTCGATAGTCGAGCCGGTCTATCGGCGTATTGAGGAAACATTTCAGGAAAACGAAAAATGTGATCTAACCGAAGTGGACTATATAAACAGCTTAGATGCTTTCACGCCGGTCAAGAAAGATTCTCCGTATTTGGAACTGCTTAGAGTTTCTTACAAACACGTGCGTGAAGTTGGTATTCAAGCGGCTTTGAACAAGCGCTATCAAGTACCCAAGCCTCGATGCGAGAGCAAAGCGGTCGCATTCAGCAGTGTCGGTCTTCTAGACCTTCGACCGGTCCTTATTATGATGATGTACGGCGTGGCTTTATCTCTAGCGATATTATTAGCGGAAATCATTGTTTTCAAAATGTGGGTAT

>BmIR75p.2

MYLWRIPLLITSDLVLARRYGDLFELVELHKPAPSGSMITTPRGFFNGSLIDMRPQKELYRRRRNLMGHHLTMANIIQDSNTTKYYLPRDDRLQLHHDAVAKVCWVFAKVAFELLNATPRYIFSYRFGYKVNGQWSGMVRDIGDNKADLEYDLHYSLSLKGTSSMHFKERLEIITYTDLVAPMRMRFVFRQPPLAYVANIFALPFSTGVWMAVAIASIASTVTLYITSLWEIRIEKNPTQLDGSIGDALLLTMSAVTQQAVIEPRRHQGLFAFHSIVEPVYRRIEETFQENEKCDLTEVDYINSLDAFTPVKKDSPYLELLRVSYKHVREVGIQAALNKRYQVPKPRCESKAVAFSSVGLLDLRPVLIMMMYGVALSLAILLAEIIVFKMWV

>OBP47

ATGGTCGCAGTTTTGATATTGTTCACTGTCTTTCCAACACTGATTTTGTGTTCTGGTCAAGGCAATATTCAATTACTAGAAGATGAAATATCTATCGCACTTAAGTCATGTGCTATTACGGATTTGGGAGCCGGTACTCGACAAAAAAGATCTAATAATTTATATAATAATGGTGGTTTAGACGGCAACTCGAAGCAAACGTCAAAGAAATATATTCTCGAAAGACGTAACACTGATGGGAGACGTGAGCAGATTTACAAAATTAATACAACTGATGACGATTTTGAAAACGATGCACCTAATGAAAAACAATGGTTTAGTTTTTCGTCACCACAAATGGGAGGCGCCCATCATTACAACTCTAGAAAACTGTATAGGGCGAAACGCAACGAACCACTTTTTAATAAATCTGACACTGACCAGTGCCTTAGTCAATGTGTGTTTGCCAATTTACAAGTGGTGGATCCGCATGGTATCCCACGTGAAGCTGAATTGTGGAACCTTGTGCAGTCTTCAATCACATCCCAACAGTCACGTGCTGCTTTACATGACCAAATACATGCTTGCTTTCAAGAACTACAATCCGAAACCGAAGACAACGGATGTTCATATTCAAACAAACTAGAAAAATGTCTCATGTTGCGCTTCGCTGAGAGAAAAGAGAAGAATATGAAATCAAGAAATTCTAACTAG

>OBP47

MVAVLILFTVFPTLILCSGQGNIQLLEDEISIALKSCAITDLGAGTRQKRSNNLYNNGGLDGNSKQTSKKYILERRNTDGRREQIYKINTTDDDFENDAPNEKQWFSFSSPQMGGAHHYNSRKLYRAKRNEPLFNKSDTDQCLSQCVFANLQVVDPHGIPREAELWNLVQSSITSQQSRAALHDQIHACFQELQSETEDNGCSYSNKLEKCLMLRFAERKEKNMKSRNSN

>OBP48

atgtgtttcgctagatactcttattattactttgtgttcctctgcctactggctctggagtgttacacactgaattgccgcacagatggtggtccgaaagaatctgaacttaagaaaatttatacaaagtgtttgaagattcaagaggataagacatctaacagttctcgaagcaatagccaagcttggaataagccacagagaaacaatcataaccaaagacgtgaccaagaaagagatcggtctggtaacgacgatagaaggagaaacagaaatgacgagattaataataatgatggtagaatgggttacaatgaaaacagaatgagcagtaatgatagaatgagaggcaacgatggaatgagtggtaatgaacgaatgagtggtaaaatgggtgatcggtacgatatgatgggcgaacgtcctgagagaggcagtggagatcatttcaatgctcgtaatgattttatgggagacaaatttggggaaatgtcagcgtacggttcctatcaatcttctacgacacccccgaggaggtacaaacgtgaaagacgcattgagaactcaggacatcggagccaatttaatcccatcagtcagaaatctgacgatgacgattctttcaatgacgaaaaggattctaatgaaaataaaacgaacgatgaaatgggcagcaaggaatgtgcaatgcactgtttcttagagaacttgaaaatgaacggagaagacggaatgccggatagatatttggtgaatcatgcattaactaaagaggtaaaaaatgaggatttaagagattttttgcaagagtccgtcgaagaatgttttcaaattttggataacgaaaacaccgacgacaaatgtgacttctcaaggaatctgctgatgtgcttgtctgagaagggaagagcaaattgtgacgattggaaagacgatttgcaactttga

>OBP48

MCFARYSYYYFVFLCLLALECYTLNCRTDGGPKESELKKIYTKCLKIQEDKTSNSSRSNSQAWNKPQRNNHNQRRDQERDRSGNDDRRRNRNDEINNNDGRMGYNENRMSSNDRMRGNDGMSGNERMSGKMGDRYDMMGERPERGSGDHFNARNDFMGDKFGEMSAYGSYQSSTTPPRRYKRERRIENSGHRSQFNPISQKSDDDDSFNDEKDSNENKTNDEMGSKECAMHCFLENLKMNGEDGMPDRYLVNHALTKEVKNEDLRDFLQESVEECFQILDNENTDDKCDFSRNLLMCLSEKGRANCDDWKDDLQL

>Bmjhe5

ATGTCGAATACATTTTATTTAATTACAGTTTTAATTCTTGTATTAAATACAATAAATGCAAAAGTTATCATAACATCTAATGGGCCTGTTAGAGGAAGTCTCGTTACTCACGAGAACGTGTCATATATTGCTTATCGTGGCATTCCTTATGCGGAAAATCCTACAGGTGCATTACGATTTCAGCCTCCAGTTACCAAAAAAAAATGGACTCACGTTTTAAATGCTTCTTCAAACGGTCCAGTTTGTATTCAACCAGAGGGTGCCTACAAGAAACATCAAATGGGAGAAGATTGTTTAAGAATCAATGTCTACGTTCCCGTAAAAAAAACAAACACATCCCACTTACCTGTATTTGCTTTTATACATGGGGGAGCATATAGAATTTTATCTAAAGATTCTGATGTTATTTACGACCCGTATTTCTTTATGAAAAAGGGAATAATAGTAGTTACAATGAACTACAGATTGGGAGCTTTAGGATTTTTATCGCTCAAAACACCTGGAGCATCTGGAAATAATGGACTAAAAGATCTTGTACTAG

>Bmjhe5

MSNTFYLITVLILVLNTINAKVIITSNGPVRGSLVTHENVSYIAYRGIPYAENPTGALRFQPPVTKKKWTHVLNASSNGPVCIQPEGAYKKHQMGEDCLRINVYVPVKKTNTSHLPVFAFIHGGAYRILSKDSDVIYDPYFFMKKGIIVVTMNYRLGALGFLSLKTPGASGNNGLKDLVL

>BmCYP4C1

aaaaaaatatttcaggaaaagctttacgaagaaattactaaaataatcggtgataaagacagatgtgttacgaacgaagatttcaaacaaatgccctacttggatatggtcttcaaggaagttttacgtctttttccaattggtagcatgctgcaaaggactattaatgaaaatattcggataagttctgggacactaccagcagggtgttccttggtagcaccgatatatcatttgcatcgcgactctcgtttctggactaggcccgacgatttcgatcccgagagattcaatccagttaatacgaagttgagacaaccgaactgctatattccttttagtttaggacccatggactgtttgggaagatacttcggtactaggttagtaaaaatgatttgcgttcgagtactaagagagttcgaagtgacaacatctagaacatacaaggatctcaaactatcgatagctatatctgtgtctccattggatgggttcccagttaaattacatcccaggaaaattaaagagaaacagtaa

>BmCYP4C1

KKIFQEKLYEEITKIIGDKDRCVTNEDFKQMPYLDMVFKEVLRLFPIGSMLQRTINENIRISSGTLPAGCSLVAPIYHLHRDSRFWTRPDDFDPERFNPVNTKLRQPNCYIPFSLGPMDCLGRYFGTRLVKMICVRVLREFEVTTSRTYKDLKLSIAISVSPLDGFPVKLHPRKIKEKQ
