# Supplementary material for: Evidence of peripheral olfactory impairment in the domestic silkworms: insight from the comparative transcriptome and population genetics
Source: BMC Genomics. 2018 Nov 1;19:788. doi: 10.1186/s12864-018-5172-1 (PMC6211594; doi:10.1186/s12864-018-5172-1)
Supplement: Supplementary file 11 — Table S9. The sex-biased genes in the domestic and wild silkworms. The putative functions were from annotations through BLAST against nr database in NCBI, and the detailed information of BLAST best hits was in Additional file 4: Table S4, Supporting information. (DOCX 16 kb) [file 12864_2018_5172_MOESM11_ESM.docx]

| Gene name | D_M | D_F | W_M | W_F | Putative function |
| --- | --- | --- | --- | --- | --- |
| *BmOR1* | 330.87 | 0.08 | 910.87 | 0.29 | odorant receptor |
| *BmOR19* | 0.05 | 28.63 | 0.35 | 38.39 | odorant receptor |
| *BmOR3* | 198.56 | 0.22 | 557.62 | 0.28 | odorant receptor |
| *BmOR45* | 9.98 | 60.92 | 18.46 | 106.37 | odorant receptor |
| *BmOR46* | 0.33 | 7.90 | 0.95 | 9.18 | odorant receptor |
| *BmOR48* | 1.21 | 10.23 | 13.86 | 67.18 | odorant receptor |
| *BmOR6* | 12.56 | 0.01 | 14.39 | 0.13 | odorant receptor |
| *BmOR7* | 4.86 | 0.05 | 7.65 | 0.11 | odorant receptor |
| *BmOR78* | 0.10 | 3.67 | 0.02 | 4.15 | odorant receptor |
| *BmPBP1* | 98407.92 | 6299.38 | 163541.98 | 15718.18 | pheromone-binding protein |
| *BmGSTd4* | 2539.77 | 11.85 | 3783.40 | 46.11 | glutathione transferase |
| *XLOC_002430* | 86.28 | 0.24 | 145.82 | 0.51 | aldehyde dehydrogenase |
| *XLOC_000848* | 6.40 | 0.03 | 1.56 | 0.09 | tissue factor pathway inhibitor |
| *XLOC_002859* | 0.78 | 90.33 | 2.01 | 58.71 | uncharacterized protein |
| *XLOC_008889* | 0.34 | 5.74 | 0.33 | 3.29 | probable chitinase |
| *XLOC_016312* | 0.53 | 4.73 | 0.75 | 4.95 | Unknown |
| *XLOC_021779* | 0.00 | 6.37 | 3.03 | 23.05 | Unknown |

**Table S9 The sex-biased genes in the domestic and wild silkworms**

The putative functions were from annotations through BLAST against nr database in NCBI, and the detailed information of BLAST best hits were included in Table S4, Supporting information.
